# Phenotypes and environment predict seedling survival for seven co‐occurring Great Basin plant taxa growing with invasive grass

**DOI:** 10.1002/ece3.8870

**Published:** 2022-04-30

**Authors:** Alison C. Agneray, Thomas L. Parchman, Elizabeth A. Leger

**Affiliations:** ^1^ Graduate Program in Ecology, Evolution, and Conservation Biology Department of Biology University of Nevada, Reno Reno Nevada USA; ^2^ Nevada State Office Bureau of Land Management Reno USA

**Keywords:** functional traits, local adaptation, native plants, rangelands, restoration, seed source

## Abstract

Trait–environment correlations can arise from local adaptation and can identify genetically and environmentally appropriate seeds for restoration projects. However, anthropogenic changes can disrupt the relationships between traits and fitness. Finding the best seed sources for restoration may rely on describing plant traits adaptive in disturbed and invaded environments, recognizing that while traits may differ among species and functional groups, there may be similarities in the strategies that increase seedling establishment. Focusing on three grass genera, two shrub species, and two forb genera, we collected seeds of all taxa from 16 common sites in the sagebrush steppe of the western United States. We measured seed and seedling characteristics, including seed size, emergence timing, and root and shoot traits, and compiled a suite of environmental variables for each collection site. We described trait–environment associations and asked how traits or environment of origin were associated with seedling survival in invaded gardens. Sampling seven taxa from the same sites allowed us to ask how trait–environment–performance associations differ among taxa and whether natural selection favors similar traits across multiple taxa and functional groups. All taxa showed trait–environment associations consistent with local adaptation, and both environment of origin and phenotypes predicted survival in competitive restoration settings, with some commonalities among taxa. Notably, rapid emergence and larger seeds increased survival for multiple taxa. Environmental factors at collection sites, including lower slopes (especially for grasses), greater mean annual temperatures (especially for shrubs and forbs), and greater precipitation seasonality were frequently associated with increased survival. We noted one collection site with high seedling survival across all seven taxa, suggesting that conditions within some sites may result in selection for traits that increase establishment for multiple species. Thus, choosing native plant sources with the most adaptive traits, along with matching climates, will likely improve the restoration of invaded communities.

## Graphical abstract

Using seven co‐occurring taxa from 16 collection sites, we described trait‐by‐environment associations and asked how traits or environment of origin were associated with seedling survival in invaded gardens. All taxa showed TBE associations consistent with local adaptation, and both environment and phenotypic traits predicted survival in competitive restoration settings. We found that choosing native plant sources with the most adaptive traits, along with matching climates, could improve the restoration of invaded communities.

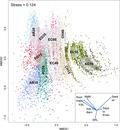

## 1 INTRODUCTION

Local adaptation is pervasive in nature, and plant populations frequently evolve phenotypes that increase fitness at their home sites (Baughman et al., 2019; Leimu & Fischer, 2008). Traits that confer advantages in a particular site may be shared by multiple species, as environmental and biotic pressures limit what ecological strategies are viable in any given locale (Grime, 2006; Helsen et al., 2012). For example, in highly seasonal environments, multiple plant species may evolve similar seed germination characteristics to time growth with resource availability (Rubio de Casas et al., 2017). Also, root allocation may increase in species growing in resource‐poor environments (White et al., 2013), resulting in co‐occurring species with similar phenotypic characteristics (Larraín‐Barrios et al., 2018). Simultaneously, character displacement occurs in co‐occurring plant taxa, as evolutionary pressure to reduce resource competition between interacting species can lead to niche differentiation in characteristics such as phenology or rooting depth (Silvertown, 2004). These concepts need not be mutually exclusive. One can consider adaptation to the environment as a first filter that results in similarity among co‐occurring taxa, with trait differentiation subsequently promoting coexistence (Grime & Pierce, 2012). This filtering results in similarities and differences in trait–environment correlations among species (DeMarche et al., 2013; Hodgins & Yeaman, 2019).

All populations exhibit varying levels of adaptation or maladaptation to their environment due to factors such as population size, gene flow, and the nature of selection (e.g., directional or fluctuating) that can influence the degree of local adaptation (Herden et al., 2019). Even in a single location, there are likely differences among species in their degree of local adaptation, with life‐history characteristics such as life span, dispersal ability, and mating system affecting the response to selection (Hodgins & Yeaman, 2019; Raffard et al., 2019). Furthermore, the introduction of invasive species can drastically change selection pressures. While some native populations may evolve rapidly in response to invaders, others may be out of equilibrium with conditions in invaded sites (Strauss et al., 2006). While many studies have examined local adaptation in plant populations (Baughman et al., 2019; Leimu & Fischer, 2008), we are aware of no study that has compared local adaptation among a suite of co‐occurring taxa across the same sites. This type of study is important not only for understanding the strength and consistency of natural selection in the wild (Siepielski et al., 2013) but also for the field of ecological restoration, which regularly applies local adaptation concepts to seed source selection (Gann et al., 2019).

Seed provenance is a key factor in the success or failure of restoration projects (Pedrini & Dixon, 2020; Shackelford et al., 2021), and the selection of locally adapted propagules can increase plant establishment (Vander Mijnsbrugge et al., 2010). In the absence of direct evidence of local adaptation, geographic proximity and climate similarity are commonly used as predictors of local adaptation, and local seeds are generally known to be more successful (local seeds outperform non‐local ones ~70–90% of the time; Baughman et al., 2019; Leimu & Fischer, 2008). Recognizing the importance of abiotic factors in shaping local adaptation, many seed transfer zones used to guide seed selection for restoration are based on climate and soil characteristics (Bower et al., 2014; Erickson & Halford, 2020). However, there can be other differences among sites that also affect trait evolution, such as interspecific interactions, invasion, or disturbance history (Baughman et al., 2019). Thus, plants from areas with similar environmental conditions may evolve different phenotypes in response to variable selection from factors other than site climates and soils (Bucharova et al., 2017; Leger et al., 2019). Restoration success could be improved by identifying seed sources that can excel at establishing in disturbed and invaded conditions through testing the field performance of multiple seed collection sites from similar climates.

Understanding the link between functional traits, environments, and seedling survival has broad implications for ecological restoration, which has increasingly sought ways to use functional trait information to increase restoration success (Carlucci et al., 2020; Garbowski et al., 2020). Is it possible to identify a set of functional traits that predict seedling recruitment for a wide range of organisms in a restoration scenario? Are these the same functional traits that show trait–environment correlations consistent with local adaptation? And, if the barriers to the seedling establishment are strong and constant enough to affect all taxa, such as competition from a widespread invasive species, do they promote convergence in successful seed and seedling characteristics? To answer these questions, one must determine: (1) whether seed sources differ in seedling survival in competitive settings, (2) whether seed sources differ in traits and exhibit trait–environment correlations, and (3) if the same traits or collection site environmental variables predict seedling survival across taxa. Comparing trait–environment associations among co‐occurring taxa can provide insight into convergence and divergence of seedling recruitment strategies. Unlike meta‐analyses and reviews (Baughman et al., 2019; Leimu & Fischer, 2008), our approach compares multiple taxa growing in the same field sites, allowing for more direct comparisons. Furthermore, comparing trait–survival associations in competitive scenarios could identify if and how recruitment strategies differ among taxa, providing information that can be used to find promising seed sources for restoration.

Here, we consider these questions in the Great Basin Desert of the Western United States, an area that is home to one of the world's largest ongoing wildland seeding efforts (Harrison et al., 2020). Seedling establishment is a major barrier to successful restoration in this region (Leck et al., 2008; Pilliod et al., 2017). Past work seeding perennial bunchgrasses into invaded Great Basin sites has demonstrated that several fitness‐related traits are under selection in disturbed restoration sites (Herget et al., 2015; Leger et al., 2019). However, we currently lack the same understanding of the traits that increase the seedling establishment of shrubs and forbs (Walker & Shaw, 2005). For this project, we collected seeds of widespread, co‐occurring taxa from multiple functional groups, described seed and seedling characteristics, planted seeds into competitive common gardens, measured emergence and survival over a growing season, and described trait–environment–performance associations for each taxon.

Given the prevalence of local adaptation in the Great Basin, we expected to find strong trait–environment correlations and that seedling traits and trait–environment correlations would vary by taxa (Baughman et al., 2019). For example, taxa with early phenology, such as *Elymus* spp. and *Poa secunda*, may have traits that promote resource acquisition, such as fine root production and high specific root length, especially when sourced from relatively dry environments. In contrast, we expected that longer‐lived shrubs might focus on resource conservation and storage with, for example, high allocation to larger root structures in dry environments. Furthermore, we expected that while seedlings of different taxa would differ in phenotypes, some successful establishment strategies, such as rapid germination, might be shared among taxa when grown in competition with invasive annual grasses. The results of this work will result in a better picture of the trait–environment correlations that reveal the major drivers of local adaptation for a suite of taxa, along with the trait–performance associations that can be used to improve restoration seed choice for disturbed and invaded sites.

## 2 METHODS

### 2.1 Species and collection site selection

We focused on the western Great Basin, a region with few native seed sources available for large‐scale seeding (USDA NRCS, 2021). Primary drivers of local adaptation in the Great Basin include elevation, mean annual temperature, precipitation seasonality, and invasion history, and traits commonly associated with local adaptation in this region include a variety of phenological traits and plant size (Baughman et al., 2019).

To identify our target species, we conducted plant surveys in 80 sagebrush‐steppe ecosystems to choose the most commonly co‐occurring plant taxa. From a list of 187 candidate species, we selected seven of the most common native plant taxa representing a variety of life forms, including grasses, shrubs, and forbs (Table S1), and identified sites where all taxa co‐occurred. Despite the widespread distributions of many Great Basin plant species, it was difficult to find locations where the same set of species co‐occurred. Therefore, we included multiple species for some genera. Specifically, we included both *Elymus elymoides* (Raf.) Swezey and *E*. *multisetus* M.E. Jones, which co‐occurred at eight sites, as well as five different closely related species of *Erigeron* (Noyes, 2000). For analysis, we considered the performance of *Erigeron* and *Elymus* at the genus level. Other species included the shrubs *Artemisia tridentata* Nutt. and *Ericameria nauseosa* (Pall. ex Pursh) G.L. Nesom & Baird, the grasse *Achnatherum thurberianum* (Piper) Barkworth and *Poa secunda* J. Presl, and the forbs *Chaenactis douglasii* (Hook.) Hook. & Arn., *Erigeron aphanactis* (A. Gray) Greene, *E*. *filifolius* (Hook.) Nutt., *E*. *linearis* (Hook.) Piper, *E*. *bloomeri* A. Gray, and *E*. *eatonii* A. Gray.

**FIGURE 1:**
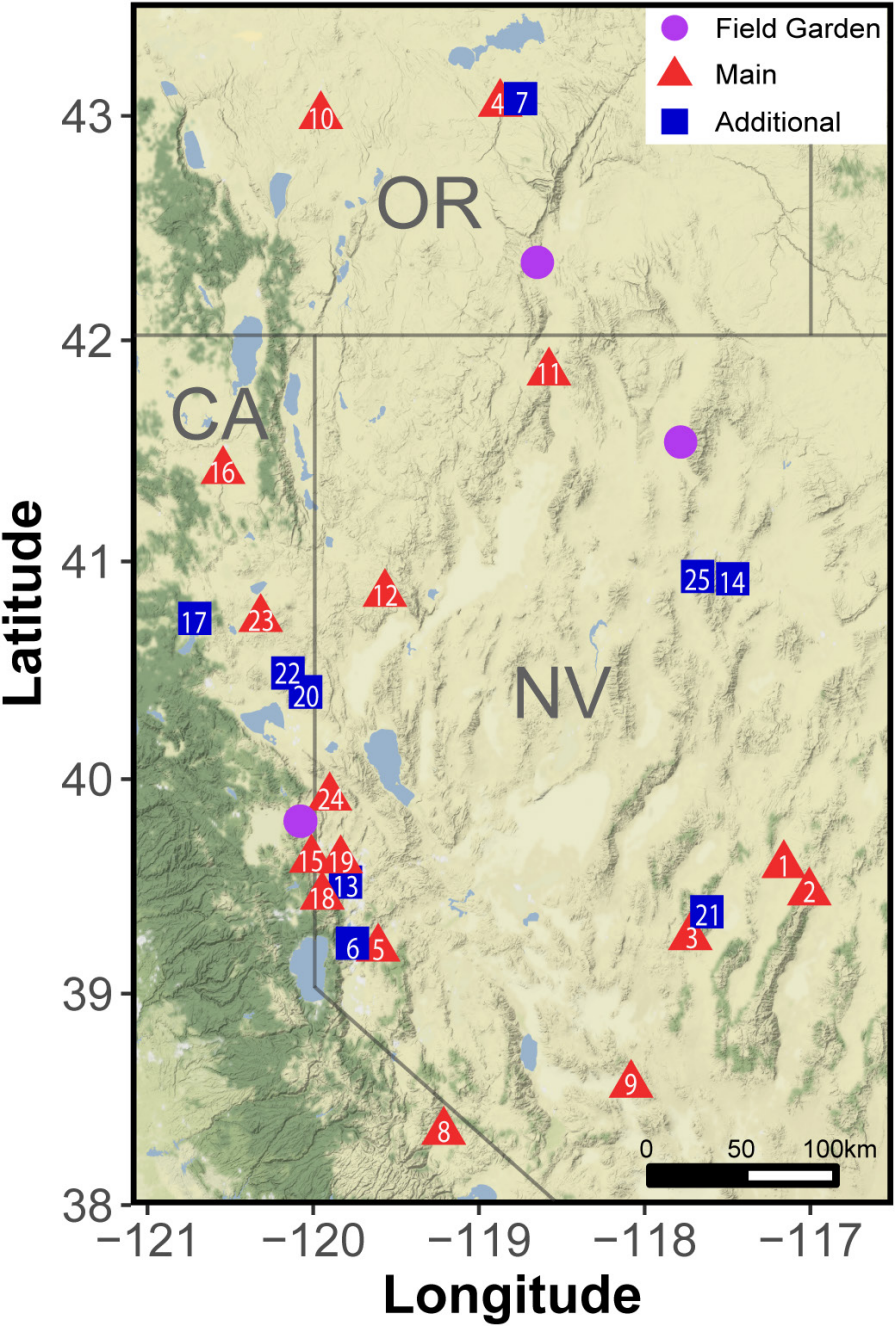
Seed collection locations (red triangles, blue squares) and common garden locations (purple circles). Sixteen main community sites (red triangles) were included for all taxa, and nine additional sites (blue squares) were only included as additional collection sites for grasses. Numbers correspond with site information in Table S2

We identified 16 collection sites where all taxa occurred and collected additional seeds for the more common grass species from nine sites, resulting in 131 seed sources from 25 seed collection sites (Figure 1, Table S1). Sites were primarily low elevation, sagebrush steppe communities, with average annual precipitation between 190 and 388 mm and elevation ranging from 1275 to 2400 m for all sites (PRISM Climate Group, 2004; Table S2). We bulk‐collected mature seeds from a minimum of fifty individual plants per taxon from each of these sites between 25 May and 11 November 2017. In addition, we made supplementary collections of *A*. *tridentata* between 18 November and 3 December 2018.

### 2.2 Environmental data

To characterize climate, soils, and site characteristics for each collection site and common garden location, we acquired environmental data from the PRISM Data Explorer, SSURGO Web Soil Survey, and USGS Digital Elevation Models (PRISM Climate Group, 2004; Soil Survey Staff NRCS‐USDA, 2021; USGS, 2019; Table S2). Next, we estimated a suite of functionally relevant environmental variables associated with temperature, precipitation, landscape position, and soil characteristics using 30‐year normal climate data between 1981 and 2010 and NRCS SSURGO soil information (Table S2; Dilts et al., 2015; Redmond, 2019; Soil Survey Staff NRCS‐USDA, 2021). After z‐transforming each environmental variable and removing variables with correlations (|*r*| >0.6) to reduce multicollinearity, we retained 13 variables for analyses (Table S3). These environmental variables included: mean annual precipitation (MAP), elevation (Elev.), slope, northerliness (Nor.; degrees deviation from true north, 0°), easterliness (East.; degrees deviation from east, 90°), heat load index (Ht. ld.; measure of solar radiation; McCune & Keon, 2002), soil available water‐holding capacity (SAWC), mean annual temperature (MAT), precipitation seasonality (Ppt. s.; calculated index measures rainfall seasonality; higher values indicate greater rainfall in a short period of time followed by long dry season; Walsh & Lawler, 1981), minimum vapor pressure deficit (VPD; difference between moisture in the air and the humidity saturation point; higher VPD indicates plants are under greater pressure to draw moisture from roots), annual actual evapotranspiration (AET; higher AET values indicate sites with greater plant productivity), and steepest decline of AET (largest month‐to‐month drop in AET; higher values indicate more rapid transition into drought months).

### 2.3 Experimental data collection

Using the wild‐collected seeds of multiple taxa from semi‐arid sites in the sagebrush steppe, we first planted a series of competitive common garden experiments in the field and in the greenhouse to identify the most successful seed sources. We then measured seed and seedling traits in the greenhouse to assess trait–environment and trait–performance associations.

#### 2.3.1 Comparing field performance in competitive common gardens

Field performance was measured in three common gardens within the western Great Basin (Figure 1; Oregon: 42.3493°N, −118.6472°W; Nevada: 41.5419°N, −117.7824°W; California: 39.805355°N, −120.078776°W). The garden locations are former big sagebrush steppe communities with sandy loam soils. Each experienced at least one wildfire in the past 20 years and is currently dominated by the invasive annual grass *Bromus tectorum* L. Thirty‐year average precipitation varied between 211 and 320 mm mean annual temperature varied between 9.3°C and 9.8°C (Table S2). Over the 11 months of our field experiment, cumulative precipitation was above average in California, below average in Nevada, and normal for Oregon (Figure S1C).

In September 2017, seeds were sown directly into the ground without site preparation into ten randomized blocks per taxon per site, with three to five seeds per seed collection source in each block for a total of 14,410 seeds. Using sowing techniques similar to previous projects (Leger et al., 2019, 2021), each grass seed was glued to a toothpick using Elmer's Washable School Glue to ensure monitoring precision; shrub and forb seeds were planted directly in the ground. Each field garden was monitored for emergence and subsequent survival every month, starting in December 2017. We conducted a final census on total seedling survival in each garden at the time of senescence for each taxon (May–August 2018).

#### 2.3.2 Greenhouse competition experimental design and data collection

In the field experiment, shrubs and forbs had 0–6% emergence and seedling survival, possibly due to seed dormancy, insufficient water, or low seed viability. To generate an alternative measure of relative seed source performance for these taxa, we established competition experiments in the greenhouse, where it was easier to control temperature and water availability. Only greenhouse measurements were used in analyses for shrubs and forbs. Starting in January 2019, we sowed seeds of each collection into individual pots with or without competition from *B*. *tectorum*. Pots with no competition served as a measure of seedling emergence and survival in the absence of competition, which was included as a model covariate given the possibility of low viability or dormancy in these seeds. Pots were selected to accommodate the typical seedling size of each plant (5L for *A*. *tridentata*, 0.26L for *E*. *nauseosa* and *C*. *douglasii*, and 0.16L for *Erigeron* spp.). Sixty pots for each collection site and taxon were prepared. We sowed one to three *B*. *tectorum* seeds (determined by pot size) into half of the pots. For the first 2 months after sowing, the soil surface was kept moist to encourage germination. For the remaining 5 months, the pots were watered to saturation and then allowed to dry down (watering on 3‐ to 7‐day intervals) to create periodic water‐limited conditions. Greenhouse temperatures were set to mimic the gradation from cooler‐to‐warmer temperatures common in the Great Basin: temperatures varied between 3.3 and 15.5°C from 12 January to 23 May, between 7.2 and 18.3°C from 23 May to 31 May, and between 11.1 and 29.4°C from 31 May to 27 June 2019, when the experiments were concluded. We monitored emergence and subsequent survival weekly through the end of the experiment in June 2019.

#### 2.3.3 Quantifying seed and seedling traits to assess trait–environment and trait–performance associations

Seedlings of all taxa were grown in a controlled greenhouse environment, and trait measurements were taken using methods previously employed for describing perennial grass seeds and seedlings (Leger et al., 2021). We first measured the seed mass for ten sets of 20 seeds from each seed source. Then, 80–150 individual seeds from each seed source were planted in individual containers arranged in a randomized block design, with 8–15 blocks per taxon; sample size (Table S4) was determined by seed availability and germination characteristics of each taxon. Planting media was a 10:25:65 mixture of perlite, sand, and coarse‐textured field soil from Dayton, NV.

Each planting block was watered to maintain a moist soil surface and individual containers were monitored daily for emergence. Each seedling that survived was harvested at a specific number of days following initial emergence (see Table S4 for target ages of each taxon), and roots were gently washed from the soil. The roots of each seedling were individually scanned on WinRhizo imaging software (Arsenault et al., 1995) to quantify the average root diameter and total root length. We calculated the percent of total root length of each plant allocated to either fine (<0.4 mm root diameter) or coarse (>0.4 mm) roots, with this determination based on a visual estimate of seedling root systems. Shoots and roots were separated, then dried in a 40°C oven for 48 h and weighed. For each plant, we calculated root mass ratio (RMR; root mass/total mass; higher values indicate increased allocation to root biomass) and specific root length (SRL; total root length in m/root mass in g; higher values typically indicate greater allocation to smaller‐diameter roots). Thus, we generated data on the following seed and seedling traits: seed weight, emergence timing, fine root length (FRL), average root diameter, root mass, shoot mass, RMR, SRL, total biomass, and total root length. We observed that some seed sources had greater variation in biomass and emergence timing than others, so we calculated the coefficient of variation (CV) for these two traits as additional explanatory variables since such phenotypic variation may represent bet‐hedging strategies (Gremer & Venable, 2014).

For perennial grasses, trait measurements were taken at early stages (Table S4) identified to be most effective for predicting seedling establishment in previous experiments (Leger et al., 2019; Rowe & Leger, 2011). Because we lacked this prior information for shrubs and forbs and expected high variability across early development stages (Havrilla et al., 2021), traits were measured for these taxa at two different stages and considered separately in the statistical analyses and results. The two sets of measurements for the shrubs and forbs characterize early emergence and later‐stage seedling characteristics (Table S4).

### 2.4 Statistical analysis

#### 2.4.1 Q1: Do seed sources differ in survival in competitive environments?

Analyses using seedling emergence and subsequent survival as response variables produced nearly identical results since relatively few seedlings died after emergence. Thus, for simplicity, we present only seedling survival for all experiments. First, we determined whether total seedling survival differed among collection sites and common gardens by analyzing survival on an individual seed basis (survival to the end of the experiment = 1, dead = 0) using binomial logistic regression models implemented by lme4 in R version 4.0.4 (Bates et al., 2015; R Core Team, 2021). Two separate random intercept models were used: one for grasses grown in field common gardens (hereafter, grass model) and a second for shrubs and forbs in the greenhouse common gardens (hereafter, dicot model). The grass model included garden, collection site, taxon (species or genus), block (as a random effect), and all two‐ and three‐way interactions between fixed effects. The dicot model included collection site, taxon, block (as a random effect), taxon by collection site interaction, and a viability covariate (the percent emergence for each seed source in the non‐competition treatment).

Additionally, we created generalized linear models, separately for grasses and forbs, that only included the subset of 16 locations with all seven co‐occurring taxa. These models included planting site, seed source, taxon (species or genus), block (as a random effect), and all two‐ and three‐way interactions between fixed effects. We then created a plot displaying the relative survival (calculated for each taxon as the percent survival of each seed source divided by the total survival of that taxon in each garden) from the field and greenhouse competition tests. Furthermore, to evaluate the different relationships between seed sources and survival across taxa, we built individual models for each taxon that included collection site and block (as a random effect). The significance of all models was determined with a chi‐square test, given the 0/1 distribution of survival data, which was calculated using the package car (Fox & Weisberg, 2019). Goodness of fit (R‐values) was calculated with MuMIn (Barton, 2020).

#### 2.4.2 Q2: Do taxa and seed sources differ in functional traits and trait–environment correlations?

We first determined whether collections varied in functional traits by assessing differences among collection sites and among taxa using generalized linear mixed effect models with the trait values as a response, collection site, and block (as a random effect) as predictor variables. To then visualize and quantify dissimilarity among traits across taxa, we applied non‐metric multidimensional scaling (NMDS) using the Bray–Curtis dissimilarity index to the matrix of standardized traits for all harvest ages with the R packages vegan and ade4 (Bougeard & Dray, 2018; Chessel et al., 2004; Dray & Dufour, 2007; Dray et al., 2007; Økland, 1996; Oksanen et al., 2020; Thioulouse et al., 2018). Ellipses were added using the package psych to represent 95% confidence intervals (Revelle, 2020).

Then, as an estimate of local adaptation, we assessed trait–environment associations using generalized linear mixed models. We log‐transformed all trait data except RMR to improve normality and meet model assumptions and scaled data by subtracting the mean and dividing by the standard deviation for all traits for each taxon and age at harvest (shrubs and forbs only). As several seed and seedling traits were correlated with each other, we reduced the dataset by identifying nine traits that were less correlated (|*r*| < .6); calculated across all taxa based on Pearson's product moment correlation coefficients calculated in R (R Core Team, 2021; Table S5). For shrubs and forbs, we used trait values from the stage that was most predictive of survival in competitive environments.

To examine trait–environment associations separately within each taxa, we used generalized linear mixed models to quantify trait–environment associations for each taxon, using mean trait values for each collection. In these models, collection site was a fixed effect, and planting blocks were random effects. We then calculated Pearson's product moment correlation coefficients between each trait and environmental variable for each taxon to evaluate these associations. In order to ask whether there was consistency in patterns of trait–environment associations across all taxa, we used generalized linear mixed models to quantify trait–environment associations in models that included all taxa, using mean trait values for each collection.

For grasses, we additionally tested for further evidence of local adaptation by assessing the extent to which environmental distances between collection and garden sites predicted variation in field performance (this analysis was not possible for shrubs and forbs, as those studies were conducted in a greenhouse environment). To do this, we calculated relative emergence and survival values for each seed source at each garden for each taxon. Relative emergence and survival were calculated for each seed source by dividing the mean survival by collection site by the garden mean for that taxon. These relative values were log‐transformed to improve normality. We then created a distance matrix describing the Euclidean environmental distance between each seed source and garden and asked whether this distance could predict relative emergence or survival across taxa using linear regression models. We report both relative emergence and survival for this analysis, as these life stages each provided different results.

#### 2.4.3 Q3: Do seed and seedling traits or environment of origin predict survival in a restoration environment? Do important variables vary among taxa?

To determine which seed and seedling traits or environmental variables were most predictive of survival in the competitive common garden experiments, we analyzed correlations between traits and survival (using seed and seedling traits described in the greenhouse) and between environmental variables for each collection site and survival. We used the same aggregated and scaled environment and trait data described in question 2, as well as grass survival from field common gardens and shrub and forb survival in the greenhouse competition studies.

To determine the most predictive models for survival by taxon in each experiment, we constructed the same models for each taxon separately for each common garden site and performed model selection and multi‐model averaging using a genetic algorithm in the glmulti R package using AIC for model comparison (Calcagno & de Mazancourt, 2010). For both environmental and trait models, we removed any variable with a VIF greater than 4 (Fox & Weisberg, 2019). Reported coefficient estimates were calculated based on the subset of models, which yielded 95% of the total evidence weight. Pearson's correlation coefficients were then calculated for environmental variables and relative survival, as well as for trait variables and relative survival.

## 3 RESULTS

### 3.1 Q1. Do seed sources differ in survival in competitive environments?

Binomial logistic regression models indicated that field common gardens differed significantly from each other in total survival (χ^2^_(70)_ = 152, *p* < .0001) and grass taxa performed differently in each garden (χ^2^_(56)_ = 159, *p* < .0001). Averaged across all grass taxa, the Nevada garden had the lowest survival (15%), followed by California (26%) and Oregon (30%), which did not follow cumulative precipitation patterns across gardens (Figure S1). In the field gardens, there was a significant interaction between taxa and garden location (χ^2^_(37)_ = 99, *p* < .0001), with certain taxa performing better in different locations. *Elymus* spp. survived best in Oregon (average 59% overall; Table 1). *P*. *secunda* and *A*. *thurberianum* had the highest survival in California (average 29% and 25%, respectively), and California was the only garden where *A*. *thurberianum* survival was over 6% (Table 1). Seed source collections of *Elymus* differed in survival in all three field competition gardens. In contrast, the survival of *P*. *secunda* and *A*. *thurberianum* differed by collection source only in the California garden (Table 1). Unlike the other two grass taxa, *Elymus* spp. had a significant garden by seed source interaction (χ^2^_(32)_ = 46.7, *p* = .045), indicating that different seed collection sources performed best at each garden.

**TABLE 1:** Differences in survival among collection sites and performance gardens for each taxon

Taxa	Garden	χ**^2^**	*R* ^2^, *p*	Mean survival	Range in seed sources
*A. tridentata*	GH	34.3	.44**	27%	5–61%
*C. douglasii*	GH	41.4	.18***	21%	3–50%
*Elymus*spp.	CA	39.8	.10***	25%	6–46%
*Elymus*spp.	NV	28.0	.12*	33%	13–57%
*Elymus*spp.	OR	67.7	.16***	59%	27–87%
*E. nauseosa*	GH	47.4	.42***	57%	3–93%
*Erigeron*spp.	GH	36.5	.79**	7%	0–23%
*P. secunda*	CA	41.8	.12**	29%	14–40%
*P. secunda*	NV	20.7	NA^a^	11%	3–23%
*P. secunda*	OR	19.4	.05	31%	20–43%
*A. thurberianum*	CA	42.0	.13**	25%	4–42%
*A. thurberianum*	NV	35.4	NA^a^	6%	0–13%
*A. thurberianum*	OR	40.2	NA^a^	5%	0–13%

Note

Values reported are from binomial logistic regression models, and include test statistics (χ^2^), model fit (*R* ^2^), the significance of seed collection source differences in survival (*p < .05, **p < .01, ***p < .001), mean survival (across all collection sites), and ranges in mean survival among collection sites. Gardens are abbreviated to state names or GH (greenhouse). While all taxa were planted in every field garden, data for the shrubs and forbs in the field were not used due to low emergence.

^a^ These models did not converge (indicated with NA) due to few successful seed sources in the field tests resulting in zero‐inflated data.

**FIGURE 2:**
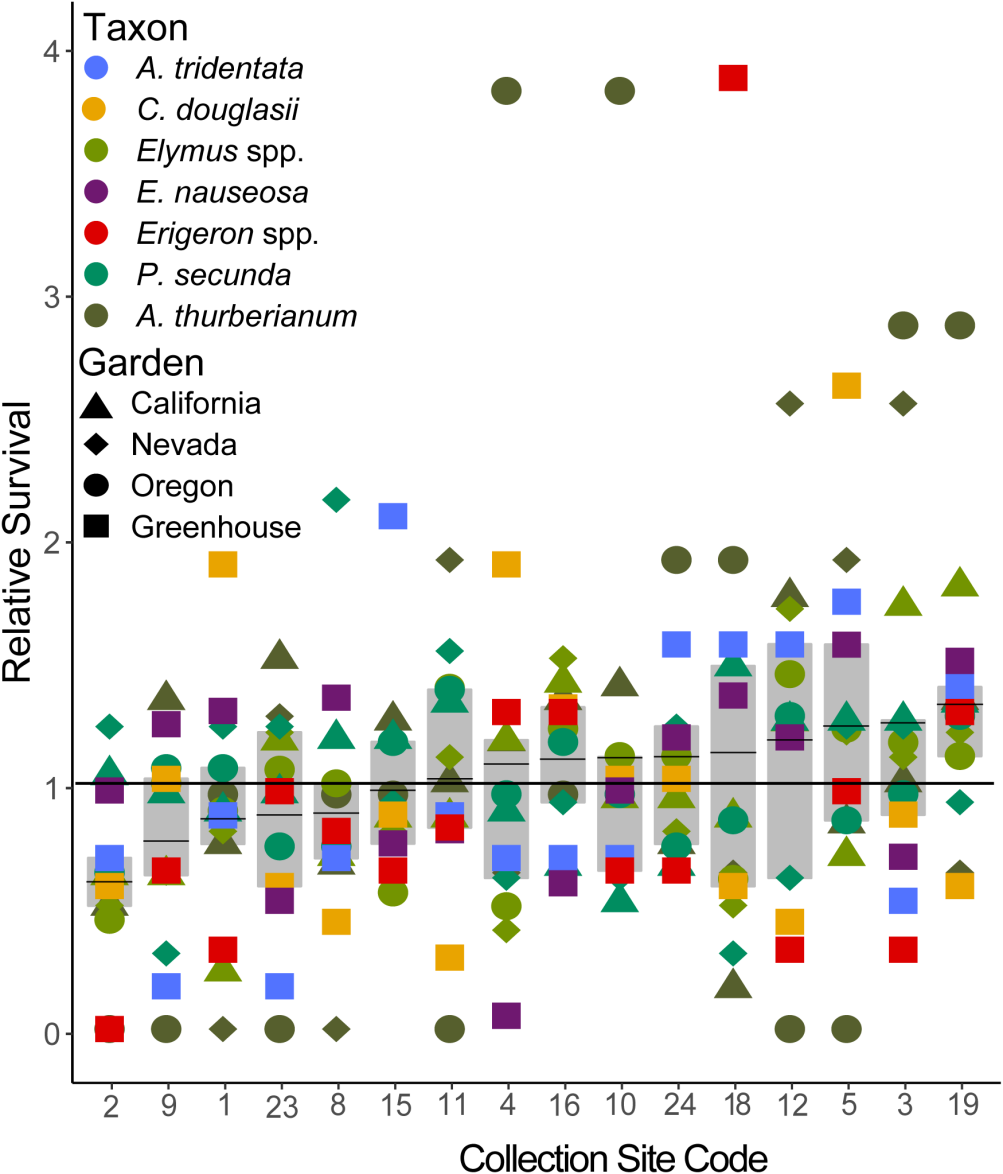
Relative survival for each seed collection source in each performance garden, ordered by mean across all test gardens. Relative survival was calculated as the percent survival of each collection source divided by total survival in each garden for each taxon (shown as different colors) and for each garden (shown as different shapes), where applicable. Values above 1 indicate that the seed source had higher performance than the average survival at each garden

In the greenhouse performance gardens, each taxon performed differently (χ^2^_(3)_ = 56, *p* < .0001). *E*. *nauseosa* had the greatest survival (57% overall) and the greatest variance among seed sources (3–93%; Table 1). *Erigeron* spp. had the lowest overall survival (7%) with relatively lower variability (0–27%; Table 1). Seed sources differed in the greenhouse competition gardens for every taxon (χ^2^_(16)_ = 57, *p* < .0001; Table 1). In the models considering only the 16 common seed collection sites, both the grass and dicot models had a significant interaction between taxa and seed source (grass: χ^2^_(51)_ = 76, *p* < .05; dicot: χ^2^_(45)_ = 101, *p* < .0001). This result indicates that, within grasses or dicots, no single seed source had uniformly high or low survival for all taxa in our common gardens. However, we observed some trends, in that there were seed sources that were generally higher or lower than average when considering survival across all gardens and taxa (Figure 2). For example, the seed source #19 had generally higher ranks across taxa in both the field and greenhouse competition, while taxa from #2 were almost all below average (Figure 2).

### 3.2 Q2: Do taxa and seed sources differ in functional traits, and do trait environment associations suggest local adaptation?

**FIGURE 3:**
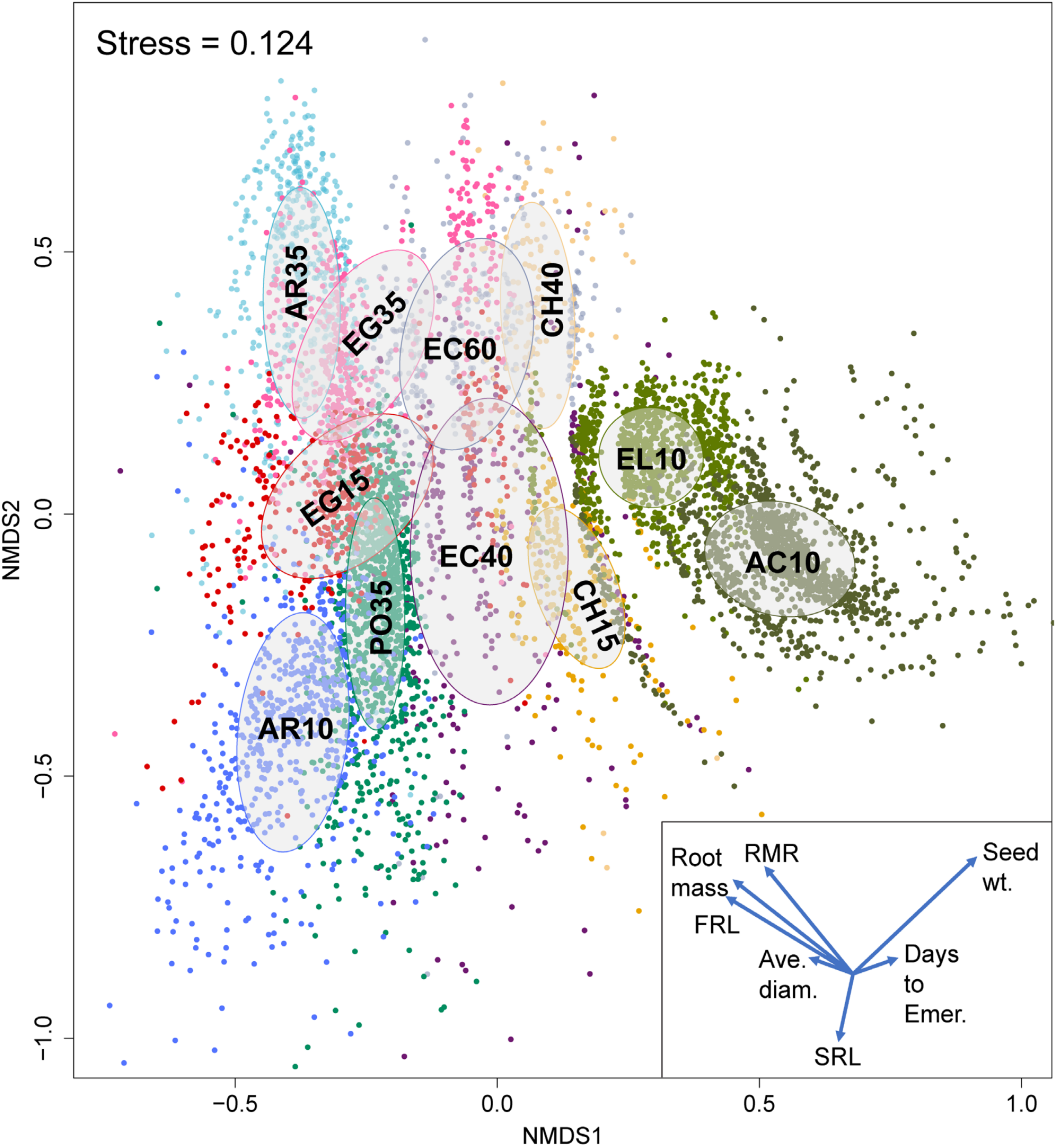
Non‐metric multidimensional scaling (NMDS) representation of seedling traits for each taxon and harvest age; ordinations were based on Bray‐Curtis dissimilarities; ellipses represent 95% confidence. Inset shows traits that exhibited a significant association with each taxon in the NMDS presented as vectors from the origin. Traits included: average root diameter (Ave. diam.), days to emergence (Days to emer.), fine root length (FRL), root mass, root mass ratio (RMR), seed mass (Seed wt.), and specific root length (SRL). Taxa included: *A*. *tridentata* harvested at ten days old (AR10), *A*. *tridentata* at 35 days old (AR35), *C*. *douglasii* at 15 days old (CH15), *C*. *douglasii* at 40 days old (CH40), *Elymus* spp. at 10 days old (EL10), *E*. *nauseosa* at 40 days old (EC40), *E*. *nauseosa* at 60 days old (EC60), *Erigeron* spp. at 15 days old (EG15), *Erigeron* spp. at 35 days old (EG35), *P*. *secunda* at 35 days old (PO35), and *A*. *thurberianum* at 10 days old (AC10)

Non‐metric multidimensional scaling revealed the separation of seedling traits among plant taxa and harvest ages into fairly distinct groups (Figure 3). For example, emergence time was different among taxa, with *A*. *tridentata*, *Erigeron* spp., and *P*. *secunda* emerging earlier and *A*. *thurberianum* and *Elymus* spp. emerging relatively later (Figure 3, Table S6). For forbs and shrubs harvested at two different ages, we observed that traits changed with different harvest ages. For example, *A*. *tridentata* switched from having a relatively high SRL (longer, thinner roots) at ten days old to low SRL at 35 days old. The stress value of 0.12 indicates a fair ordination fit. Seed sources of every taxon significantly differed in nearly all seed, phenology, biomass, and root traits (Table S6). Only four variables did not differ significantly among collection sites: days to emergence and average root diameter for *C*. *douglasii* at 15 days old, average root diameter for *A*. *tridentata* at ten days old, and RMR for *Erigeron* spp. at 15 days old (Table S6).

**FIGURE 4:**
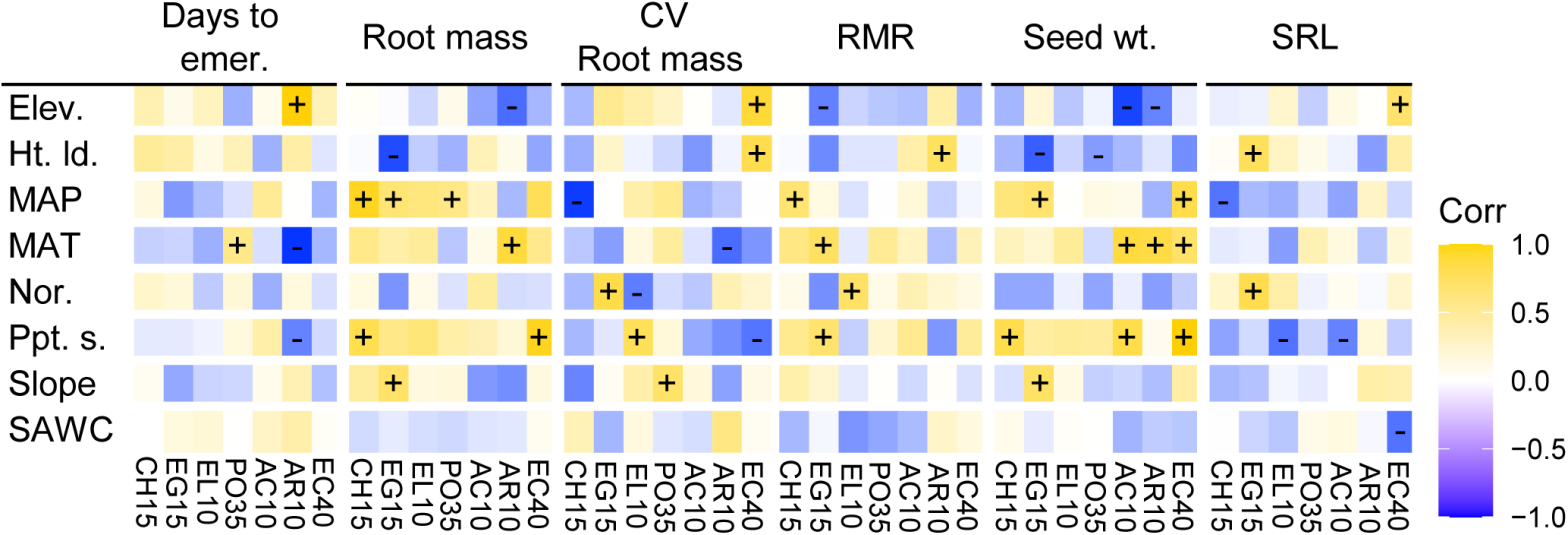
Example trait–environment correlations. The harvest age with the strongest trait–environment relationships is shown for forbs and shrubs, and we illustrate a subset of traits and environmental variables with the strongest correlations for all taxa. The color indicates the magnitude of model coefficient, brightest blue and yellow are −0.7 to 0.8, with white at 0, and “+” indicates a significant positive model coefficient, and “−” indicates a significant negative model coefficient. Environmental variable abbreviations are elevation (Elev.), heat load, (Ht. ld.), mean annual precipitation (MAP), mean annual temperature (MAT), degree of north‐facing slope (Nor.), precipitation seasonality (Ppt. s.), and soil available water capacity (SAWC). Taxa follow the same abbreviations as Figure 3

Across collection sites for all taxa, there were numerous trait–environment associations (Table S7). Several environmental variables were associated with phenotypic variation across multiple taxa, including mean annual precipitation, mean annual temperature, and precipitation seasonality (Figure 4). While not all trait–environment associations had the same directionality across taxa (Figure 4, Table S8), there were several cases where all taxa showed similar trait–environment relationships (Figure 4 and S2, Table S7). For example, across taxa, seed weights were larger when seed collection sites had greater precipitation seasonality (*F* = 16.1, *p* < .001) and greater precipitation overall (*F* = 7.3, *p* = .007; Table S7b). Furthermore, seeds tended to germinate faster when sourced from sites with low water storage capacity in the soil (*F* = 4.8, *p* = .029; Table S7d) and seeds had more variable germination timing when sourced from colder locations (*F* = 8.7, *p* = .003) and flatter sites (*F* = 19.9, *p* < .001; Table S7g).

When considered individually, each taxon had at least one trait correlated with at least one environmental variable (Figure 4). The shrubs *E*. *nauseosa* and *A*. *tridentata* had the strongest and most frequent trait–environment associations, as high as *|r|* = .85. In contrast, *Elymus* spp. and *P*. *secunda* generally had the weakest correlations between traits and environment (Figure 4; maximum *|r|* = .51 and .57; respectively), with other taxa somewhere between these extremes. While most taxa from warmer sites had earlier emergence, *P*. *secunda* showed the opposite pattern. Other relationships were more variable among taxa, with CV root mass and RMR, in particular, showing contrasting patterns among taxa in these taxon‐specific models (Figure 4, Table S8).

Finally, as additional evidence for local adaptation in grass taxa, increased environmental distance between the collection site and common garden location resulted in reductions in emergence and survival, and results varied somewhat by life stage. Specifically, relative seedling emergence was predicted (*p* = .007, *R* ^2^ = 0.06), and relative survival was weakly predicted by environmental distance (*p* = .08, *R* ^2^ = 0.03) for all three grass taxa, indicating that seeds performed better at climatically similar test gardens.

### 3.3 Q3: Do seed and seedling traits or environment of origin predict survival in a restoration environment? Do important variables vary among taxa?

**FIGURE 5:**
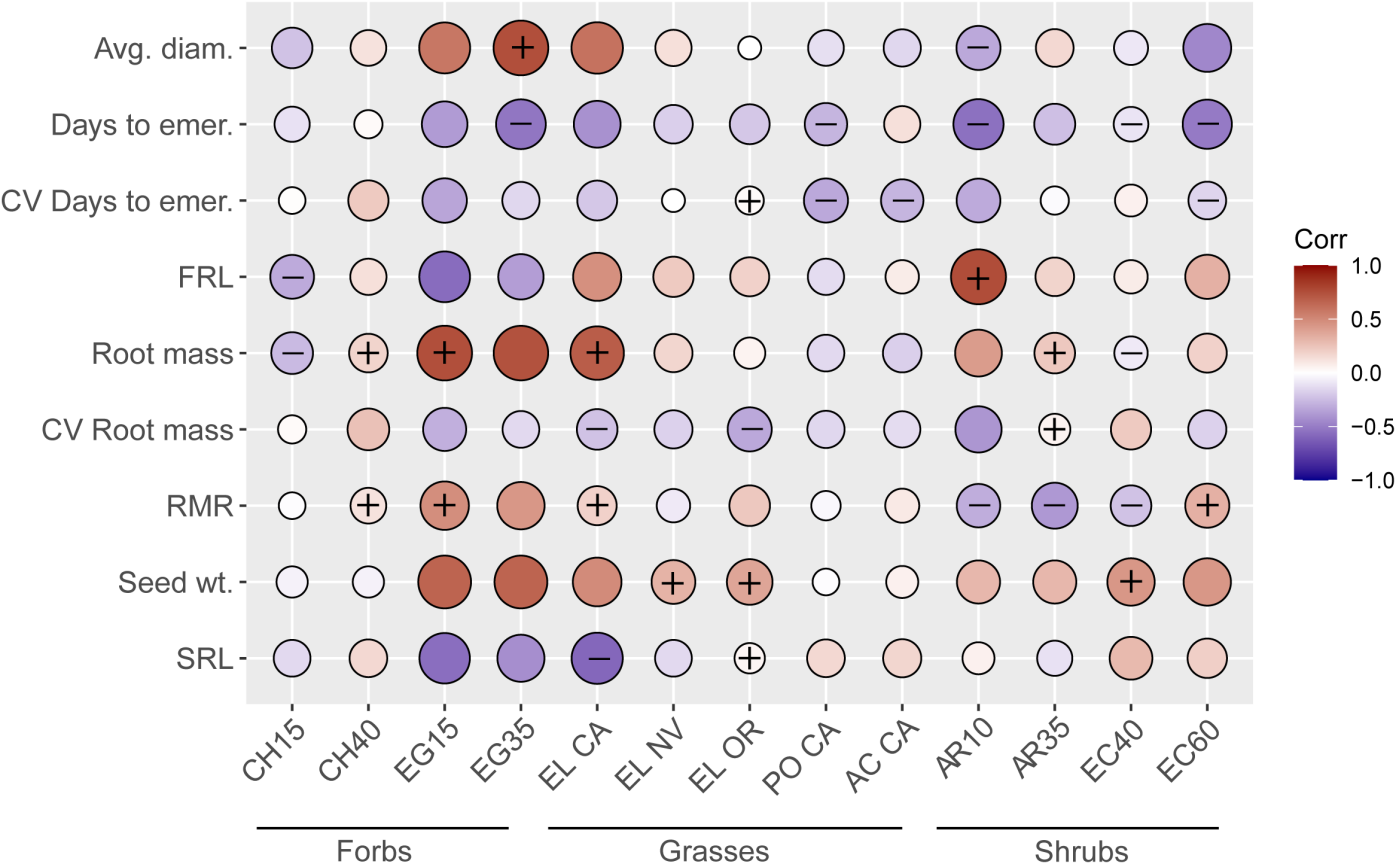
Pearson's correlations between relative survival and a subset of seed and seedling traits. Values are scaled, and the color and size of each circle indicate the strength and direction of the relationship. “+” or “−” indicates a significantly positive or negative predictive effect on relative survival for each taxon, as determined by model selection (Table S9). Traits and taxa follow the same abbreviations as previous figures

**FIGURE 6:**
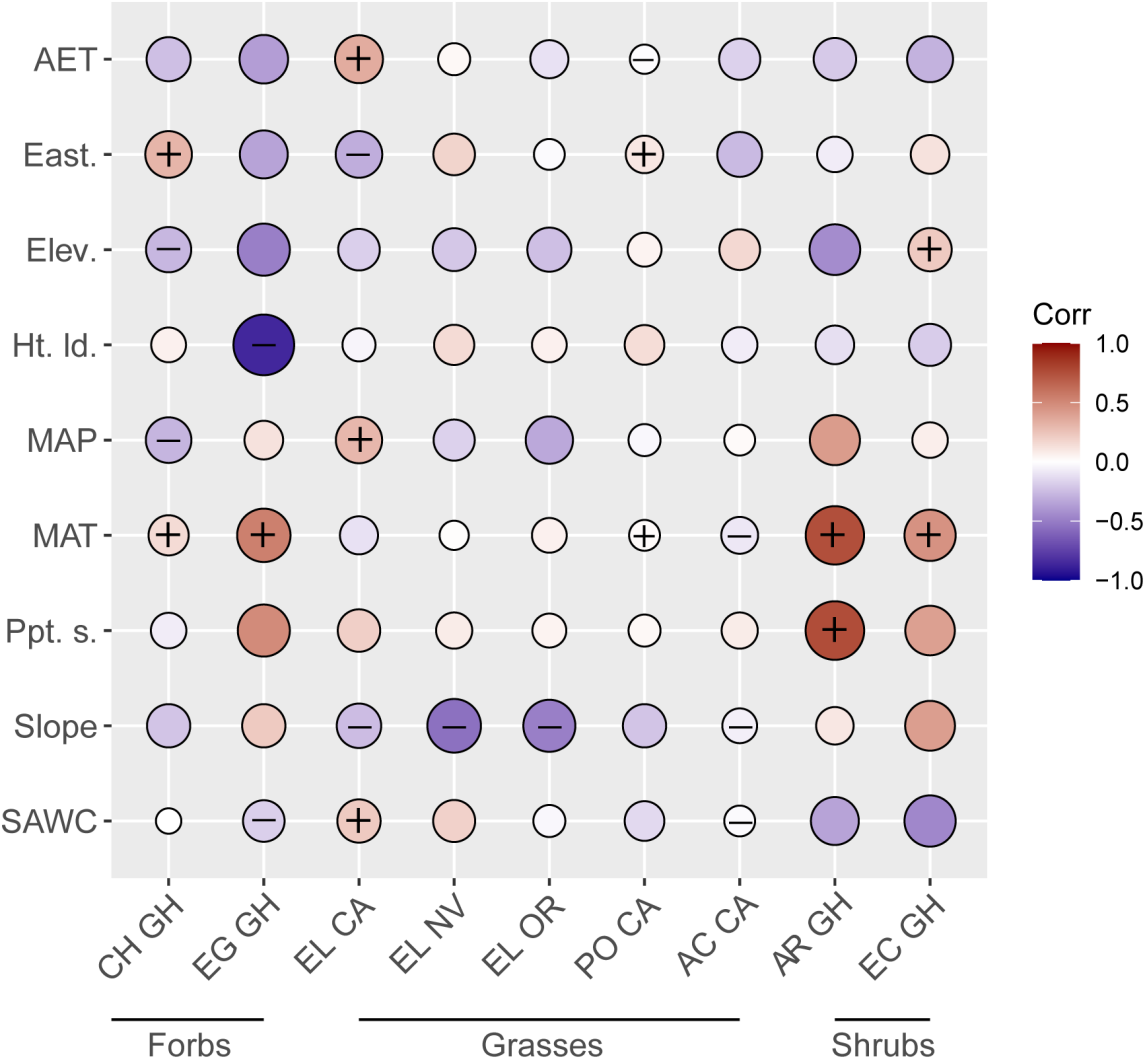
Pearson's correlations between relative survival and a subset of scaled environment variables for all seed collection sites. Color and size indicate the strength of correlation. “+” indicates a positive predictive effect, and “−” indicates a negative predictive effect on relative survival for each taxon as determined by model selection (Table S10). Traits, environmental variables, and garden locations follow the same abbreviations as Figures 3, 4, and Table 1. Additional environmental variables include average actual evapotranspiration (AET) and degree of eastern facing slope (East.)

Both traits (Figure 5, Table S9) and environment of origin (Figure 6, Table S10) were predictive of survival across all taxa and performance gardens. Models with environmental variables at the site of origin explained similar variance as trait models (average [range] *R* ^2^ for environment models = 0.29 [0–0.94]; trait models = 0.36 [0–0.83]), and model coefficients were also similar (average environment model coefficient = |0.07| ± 0.08; trait model = |0.11| ± 0.11). Overall, survival for shrubs and forbs in the greenhouse competition study was more strongly linked to environment (average [range] *R* ^2^ = 0.39 [0–0.93]) and seedling traits (*R* ^2^ = 0.48 [0–0.83]) than survival of grasses in the field (*R* ^2^ for environment models = 0.20 [0–0.59]; trait models = 0.19 [0–0.53]).

#### 3.3.1 Associations between seed and seedling traits and survival

Two traits were consistent predictors for survival across multiple taxa, as evidenced by their appearance in multiple regression models within two AIC of each other in model comparison (subsequently referred to as top models; Figure 5, Table S9). Specifically, model relationships indicated that collection sites with larger seeds that emerged earlier were most likely to survive in competitive environments. In addition, strong relationships between days to emergence, root mass, and survival were observed in multiple top models for multiple individual taxa (Figure 5, Table S9). Other traits stood out as important predictors of survival but varied in the direction of the relationship. For example, a high RMR increased survival for both forb taxa, *Elymus* spp. in two common garden locations, and *E*. *nauseosa* at the older harvest age, but the opposite was true for *A*. *tridentata* at all ages and young *E*. *nauseosa* (Figure 5, Table S9).

Similarly, a lower SRL (indicating coarse roots) was associated with higher survival for *Erigeron* spp., young *C*. *douglasii*, and *Elymus* spp. in two gardens. In contrast, there were neutral or opposite correlations for other taxa and other common gardens. For shrubs and *C*. *douglasii*, the age at harvest influenced which traits were associated with survival. Specifically, for both *E*. *nauseosa* and *C*. *douglasii*, root mass increased survival for older plants. For *C*. *douglasii* and *A*. *tridentata*, smaller diameter roots in younger plants increased survival, but this effect disappeared over time (Figure 5, Table S9).

#### 3.3.2 Associations between the environment of origin and seedling survival

Several environmental variables were important predictors of seedling survival across taxa and test gardens. Relationships between mean annual temperature, slope, and survival were in multiple top models for multiple taxa (Figure 6, Table S10). For example, the slope at the seed collection site was predictive of survival for all three grass taxa and *C*. *douglasii*, with seeds from flatter collection sites having the highest survival in competitive environments (Figure 6, Table S10). For shrubs and forbs, seeds from sites with higher mean temperatures were more likely to survive, and associations between precipitation seasonality and survival were positive for multiple taxa. Other environmental variables had more taxon‐specific effects: for example, annual precipitation, annual actual evapotranspiration, and soil available water capacity had contrasting correlations with survival both among taxa and among common garden locations for *Elymus* spp. (Figure 6, Table S10).

## 4 DISCUSSION

Understanding local adaptation across seed sources has become critical for effective restoration worldwide, particularly in the Great Basin, US, which is home to one of the largest ongoing seeding efforts in the world (Pilliod et al., 2017). Here, we present experiments that not only provide evidence of local adaptation within taxa but also compare the responses of different taxa collected from the same locations and ask whether a particular site or seed characteristics can enhance seed establishment when competing with invasive species. With this approach, we were able to quantify and compare the strength and direction of trait–environment–performance associations for multiple taxa. For grasses, we also investigated the relationship between environmental distance and performance in field common gardens. We demonstrated that both environments of origin and phenotypes predict seedling survival in competitive environments, observing both divergence among co‐occurring taxa in traits and trait–environment–performance associations as well as convergence in several characteristics that predicted survival in competitive environments. Notably, rapid emergence and larger seeds increased survival for multiple taxa. In addition, environmental factors at collection sites, including lower slopes (especially for grasses), greater mean annual temperatures (especially for shrubs and forbs), and greater precipitation seasonality were frequently associated with increased survival. Our results indicate that efforts to identify promising seed collection sources for restoration can be fruitful, even when seeking to restore a broad range of taxa and functional groups.

Local adaptation in the Great Basin is prevalent and well‐documented (Baughman et al., 2019), and the environmental heterogeneity of the region means that widespread species face divergent selection (Smith et al., 1997). Our seeds were collected from a relatively small region in the Great Basin, but nevertheless, we found trait differentiation even in seeds sourced from relatively similar environmental conditions. This result is not unexpected for species in habitats undergoing rapid changes, such as invasion or alterations in disturbance regimes, or in small populations experiencing higher rates of genetic drift (Aguilar et al., 2019; Shaw & Etterson, 2012). *P*. *secunda* and *A*. *thurberianum* were the only taxa tested here that did not show significant differentiation in survival across collection sites at two of the three field gardens tested. This lack of significance may be related to lower power to detect differences among seed collection sources due to survival of these species in these two locations (Herget et al., 2015). For all other taxa in all other gardens, it would be possible to select seed sources with the highest survival, with both environment of origin and phenotype useful for characterizing the most successful seed collections.

In the Great Basin, traits associated with plant size and phenology are frequently correlated with the environment of origin. Many species show strong effects of mean annual temperature and precipitation on size and phenology (Baughman et al., 2019; Johnson et al., 2015). Our findings concur with these observations, as we found that mean annual precipitation, precipitation seasonality, and mean annual temperature were strongly correlated with seed and seedling traits, often in similar ways for multiple co‐occurring taxa. Furthermore, we observed that multiple taxa from some sites tended to have above‐ or below‐average performance. It could be that site conditions (climatic or soil factors, invasion or disturbance history, landscape position, and gene flow) result in the evolution of similar seedling recruitment strategies. However, while natural selection may lead to trait convergence in some locations, we also found evidence of niche differentiation among seedlings of different taxa with unique phenotypes. Even at early life stages, our ordination analysis showed differentiation among taxa in seed and seedling traits. This result is consistent with possible niche segregation along various environmental gradients in light, soil moisture, and timing of resource availability (Figure 3; Martínez‐Blancas & Martorell, 2020; Silvertown, 2004).

Seed dormancy and emergence timing often dictate plant recruitment and establishment in dryland environments (Baskin & Baskin, 2014; Kildisheva et al., 2019), with fall‐germinating taxa, such as *Elymus* (Leger et al., 2019) and *Poa* (Johnson et al., 2015), emerging earlier in regions with lower fall precipitation. In our study, we found that seed sources with the earliest emergence for *Elymus*, *Erigeron*, and *Ericameria* did indeed come from drier sites (Figure 4, Table S8). Still, these correlations were relatively weak, possibly because our collection sites focused on a relatively narrow range of mean annual precipitation values (here, 190–388 mm, vs. 213–620 mm in Leger et al., 2019 and 237–1600 mm in Johnson et al., 2015). Across our collection sites, stronger relationships were found between emergence timing and temperature and elevation, with most taxa emerging earlier when sourced from warmer, lower elevation sites (Figure 4 and S2). These lower elevation sites tended to be more heavily grazed and have greater incursion of invasive species, although this was not formally measured and varies greatly from year to year. Importantly, early emergence was one of the most consistent predictors of seedling survival in competitive environments (Figure 5), which is what we would expect if there were strong selection in response to resource competition with rapidly germinating annual weeds, including the competitive *B*. *tectorum* (Ploughe et al., 2020).

Seed mass was also positively related to seedling recruitment across taxa, a result that has been found for other species across the globe (Shackelford et al., 2021). Seed mass is a complex trait, however, as it may be a proxy for other seed traits like embryo size or seed shape (Barak et al., 2018; Mukherjee et al., 2019). In addition, while seed size is generally known to have moderate heritability, it is also known to be strongly affected by the maternal environment (Baskin & Baskin, 2014). We found that plants growing from sites with greater precipitation seasonality, higher overall precipitation, and higher temperatures frequently grew the largest seeds and had the largest seedlings, similar to findings in the Colorado Plateau (Balazs et al., 2020). In contrast, sites with greater heat loads consistently produced lower seed weights across all taxa. These results could represent either maternal effects, genetic effects, or a combination of both (Baskin & Baskin, 2014). The long‐lived nature of many Great Basin perennials makes it extremely challenging to reduce maternal effects by growing species in common environments before conducting trait comparisons. However, given the importance of seed weight for seedling recruitment, such investments are warranted to understand the relative effects of genetic vs. maternal environment effects for this and other phenotypic traits.

There is a trade‐off between being an effective resource competitor and conserving resources, which impacts a species’ approach to competition (Garbowski et al., 2020; Reich, 2014). Traits associated with rapid resource acquisition (e.g., high SRL, greater root mass, early emergence) can be highly effective in competitive restoration environments (Collins et al., 2016; Leger et al., 2019). Our findings mostly aligned with these past studies: acquisition traits including greater root biomass and earlier emergence were significantly associated with survival for most taxa (Figure 5). However, resource conservation traits like low SRL and larger root diameters increased survival for *Erigeron* spp. Contrary to our predictions, this was not the case in the shrub taxa. Root allocation, measured as RMR, was found to predict survival for several taxa. While most taxa had higher survival when they had greater root investment, to our surprise, *A*. *tridentata* had greater survival when allocating more to aboveground biomass in competitive conditions (Figure 5). Interestingly, root allocation has been found to be an inconsistent predictor of native plant performance in response to competition in previous work (Garbowski et al., 2020), and the relationship between aboveground allocation and survival in *A*. *tridentata* deserves more study.

Finally, while we observed many trait–environment relationships consistent with local adaptation, not all traits strongly associated with environment were also associated with survival in competitive environments for all taxa (Figure 6). For instance, for most taxa, seed weight was closely tied to several environmental variables, including mean annual temperature and precipitation seasonality. For most taxa, seed weight was an important predictor of survival, but for others (*C*. *douglasii*, *P*. *secunda*, and *A*. *thurberianum*), it only weakly predicted survival. Furthermore, heat load (i.e., greater sun exposure) on south‐facing slopes was correlated with high SRL (longer, thinner roots) for six taxa. Still, SRL had more variable effects on seedling survival, including changes in the importance of this trait among gardens for *Elymus* spp. (Figure 6 and S4). This result might indicate that some plants and populations will be especially vulnerable to invasive species if the traits under selection for optimal local performance (i.e., slower emergence at sites with greater precipitation and higher elevations) may hinder their performance against annual invaders. Field tests would be needed to understand if and how seedlings from these environments could balance selection pressures from invasive species and environmental factors.

## 5 CONCLUSION

We found widespread evidence for trait evolution in response to environmental conditions among co‐occurring taxa, with unique phenotypes and trait–environment correlations among seedlings, along with evidence for convergence in response to competition with invasive annual species. With abundant variation among wild populations in almost all traits, our work supports the idea that traits and environmental characteristics can be used to find particular locations that would be excellent candidates to source seeds for use in seeding disturbed and invaded environments. Experiments like these can have immediate management impacts. Based on our results, we have begun working with our state and federal partners to do large‐scale collecting of seeds from our most promising seed sources, including focusing on collecting multiple taxa from sites with multiple above‐average seed collections. Furthermore, our work has identified a set of traits and environmental conditions that could be used to inform seed choice of other less‐studied taxa in this region. As more studies link trait–environment–performance associations across ecosystems, we may finally understand the factors affecting native species recruitment in disturbed systems.

## CONFLICT OF INTEREST

None declared.

## AUTHOR CONTRIBUTIONS

**Alison C. Agneray:**Data curation (lead); Formal analysis (lead); Investigation (lead); Methodology (lead); Project administration (supporting); Validation (lead); Visualization (lead); Writing – original draft (lead); Writing – review & editing (lead). **Thomas L. Parchman:** Conceptualization (equal); Formal analysis (supporting); Funding acquisition (lead); Project administration (supporting); Supervision (supporting); Visualization (lead); Writing – review & editing (supporting). **Elizabeth A. Leger:** Conceptualization (lead); Formal analysis (equal); Funding acquisition (lead); Methodology (lead); Project administration (lead); Resources (lead); Software (lead); Supervision (lead); Validation (supporting); Visualization (supporting); Writing – original draft (supporting); Writing – review & editing (supporting).

## Supporting information

Figure S1

Figure S2

Table S1

Table S2

Table S3

Table S4

Table S5

Table S6

Table S7

Table S8

Table S9

Table S10

## Data Availability

The data files are available on the Dryad Digital Repository at https://doi.org/10.5061/dryad.ttdz08m1m.
