# Supplementary figures and images for: Phenotypes and environment predict seedling survival for seven co‐occurring Great Basin plant taxa growing with invasive grass
Source: Ecol Evol. 2022 Apr 30;12(5):e8870. doi: 10.1002/ece3.8870 (PMC9055296; doi:10.1002/ece3.8870)

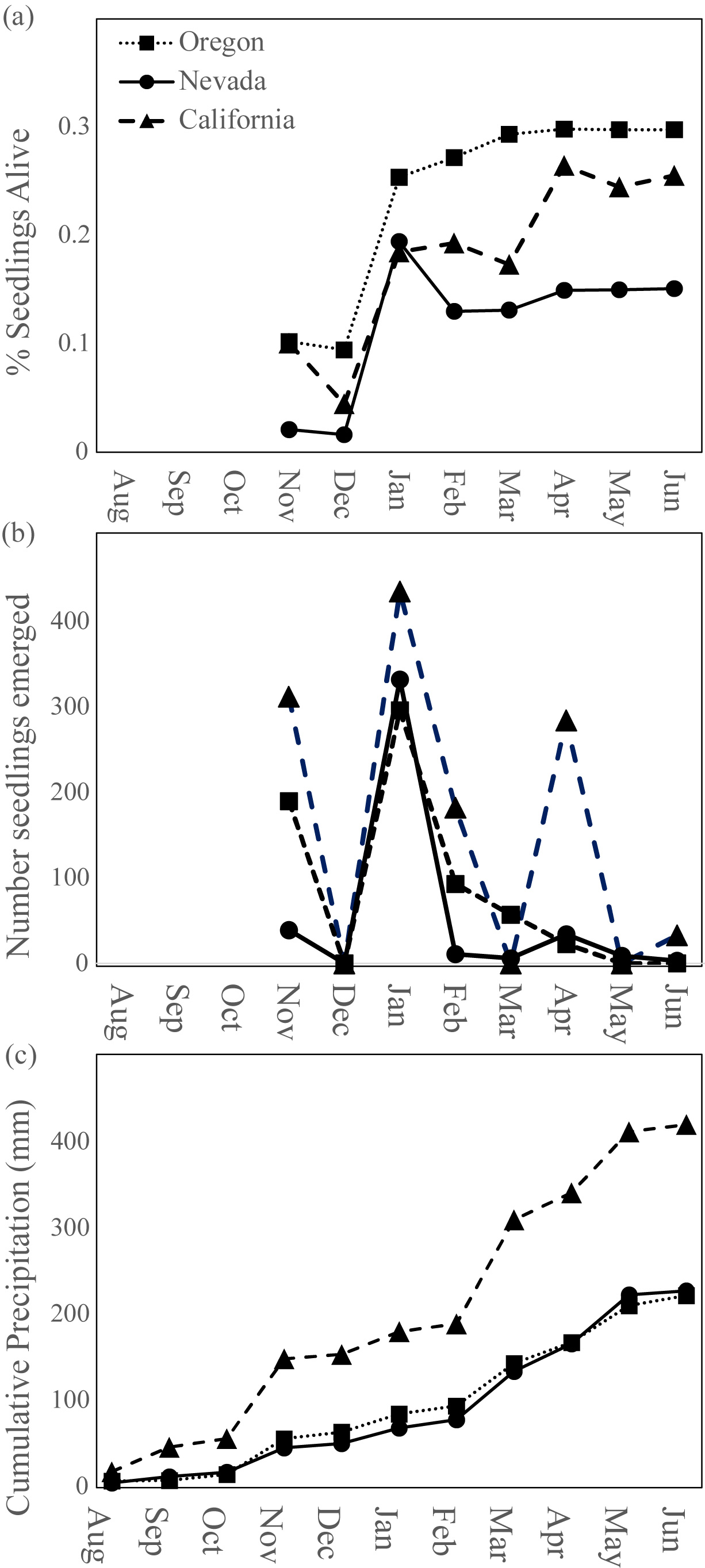

Supplement: Supplementary file 1 — Figure S1 [file ECE3-12-e8870-s011.jpg]

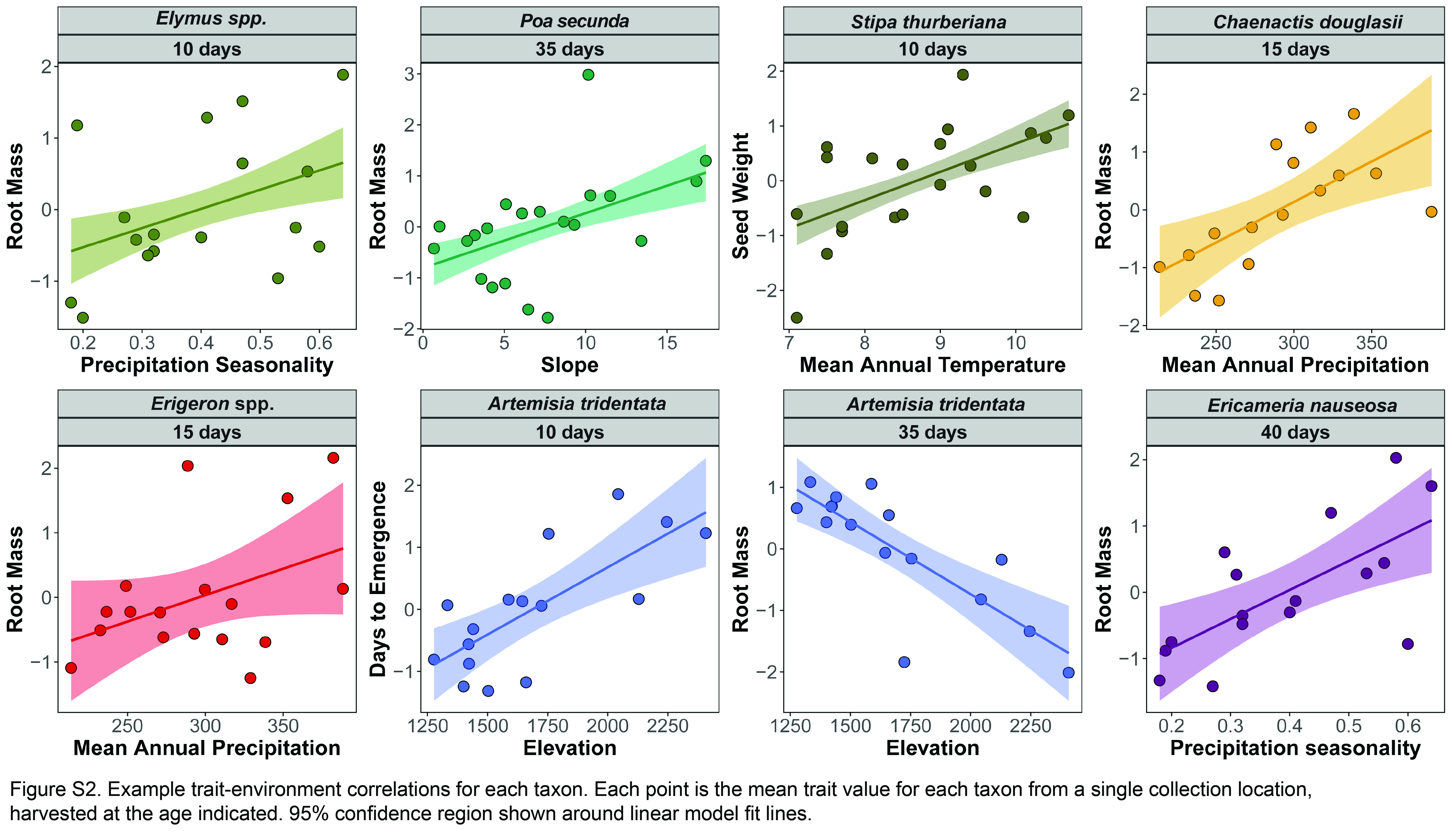

Supplement: Supplementary file 2 — Figure S2 [file ECE3-12-e8870-s007.jpg]
