# Supplementary material for: Phenotypes and environment predict seedling survival for seven co‐occurring Great Basin plant taxa growing with invasive grass
Source: Ecol Evol. 2022 Apr 30;12(5):e8870. doi: 10.1002/ece3.8870 (PMC9055296; doi:10.1002/ece3.8870)
Supplement: Supplementary file 3 — Table S1 [file ECE3-12-e8870-s012.pdf]

Table S1. Native plant taxa included in these experiments, including their functional group and the number of seed lots collected.

Taxa	Functional group	No. Seedlots
Artemisia tridentata (AR)	Perennial shrub	16
Chaenactis douglasii (CH)	Biennial/perennial forb	16
Elymus elymoides and *multisetus* (EL)	Perennial bunchgrass	17
Ericameria nauseosa (EC)	Perennial shrub	16
Erigeron filifolius, linearis, bloomeri, aphanactis, and *eatonii* † (EG)	Perennial forb	21†
Poa secunda (PO)	Perennial bunchgrass	24
Achnatherum thurberianum (AC)	Perennial bunchgrass	21

† Four sites had multiple co-occurring *Erigeron* species that were collected separately.
