# Supplementary material for: Phenotypes and environment predict seedling survival for seven co‐occurring Great Basin plant taxa growing with invasive grass
Source: Ecol Evol. 2022 Apr 30;12(5):e8870. doi: 10.1002/ece3.8870 (PMC9055296; doi:10.1002/ece3.8870)
Supplement: Supplementary file 4 — Table S2 [file ECE3-12-e8870-s010.pdf]

Table S2. (a) Abiotic variables associated with each main community site, (b) additional site, and (c) common garden site. Variables include: species collected, degree of east facing slope (East.), elevation (Elev.), heat load, (Ht. ld.), mean annual temperature (MAT), mean annual precipitation (MAP), degree of north facing slope (Nor.), precipitation seasonality (Ppt. s.), slope, and soil available water capacity (SAWC). In addition to these variables, we also included average actual evapotranspiration (AET), minimum vapor pressure deficit (Min. VPD), and monthly value of steepest decline in actual evapotranspiration (SDAET) not shown here, available in Dryad data.

**A. Main Community Site**	**Species**	**East. (degree)**	**Elev. (m)**	**Ht. ld.**	**MAT (°C)**	**MAP (mm)**	**Nor. (degree)**	**Ppt. s.**	**Slope (degree)**	**SAWC (cm)**
1. Austin Highway	All	167	1754	0.97	8.4	213.8	77.0	0.20	3.2	132
2. Austin Summit	All	155	2408	1.03	7.7	388.4	115.2	0.32	17.4	136
3. Bald Mountain Canyon	All	68	2245	0.97	7.1	299.8	158.2	0.19	7.7	112
4. Buena Vista	All	38	1277	0.92	8.1	271.0	127.9	0.29	2.7	126
5. Dayton Hill	All	107	1400	0.92	10.4	273.1	17.1	0.60	6.5	107
8. East Walker	All	7	2043	0.88	7.5	292.9	82.7	0.53	9.3	113
9. Finger Rock	All	81	2129	0.89	8.5	232.5	9.5	0.18	8.7	90
10. Grey Butte	All	45	1587	0.87	7.5	251.8	45.0	0.27	5.1	116
11. Highway 140	All	45	1423	0.94	9.0	236.6	135.0	0.32	1.0	118
12. Jones Canyon	All	115	1440	0.97	9.3	310.8	155.0	0.41	4.0	113
15. Long Valley	All	53	1660	0.91	9.0	338.6	37.4	0.58	5.1	112
16. Modoc	All	135	1333	0.94	8.5	316.9	45.0	0.40	0.7	163
18. Patagonia	All	65	1421	0.81	10.7	288.7	24.8	0.47	13.4	107
19. Peavine	All	51	1724	0.89	9.1	352.8	38.7	0.64	6.1	125
23. Spanish Springs	All	94	1645	0.92	7.5	249.1	3.8	0.31	3.6	160
24. Virginia Mountains	All	55	1503	0.94	9.6	329.0	145.0	0.56	11.5	96
**B. Additional Site**	**Species**	**East. (degree)**	**Elev. (m)**	**Ht. ld.**	**MAT (°C)**	**MAP (mm)**	**Nor. (degree)**	**Ppt. s.**	**Slope (degree)**	**SAWC**
6. Deadman Creek	AC, EG, PO	66	1677	0.77	9.4	382.2	24.4	0.69	16.8	91
7. Diamond Crater	PO	48	1295	0.92	7.9	262.3	138.4	0.27	5.7	100

13. Keystone Trail	AC, PO	124	1437	1.00	10.2	302.5	145.8	0.51	7.2	107
14. Kramer Hill	PO	5	1430	0.93	9.9	235.8	84.8	0.35	2.6	143
17. North Eagle Lake	AC, PO	27	1680	0.93	7.1	353.7	116.6	0.45	4.3	150
20. Skedaddle Spring	PO	72	1655	0.90	7.8	305.0	18.4	0.48	5.3	118
21. Smith Creek	AC, PO	81	2050	0.98	7.7	250.0	170.8	0.18	10.3	120
22. Smoke Creek	EL	90	1428	0.95	8.7	189.9	0	0.47	0.9	128
25. Water Canyon	AC, PO	89	1730	0.86	10.1	315.0	1.3	0.32	10.2	136
**C. Common garden site**		**East. (degree)**	**Elev. (m)**	**Ht. ld.**	**MAT (°C)**	**MAP (mm)**	**Nor. (degree)**	**Ppt. s.**	**Slope (degree)**	**SAWC**
California Garden		22	1515	0.93	9.8	320.0	68.2	0.52	2.6	103
Oregon Garden		0	1323	0.92	9.7	211.9	90.0	0.36	1.9	122
Nevada Garden		174	1324	0.95	9.3	260.5	83.7	0.33	2.2	146
