# Supplementary material for: Phenotypes and environment predict seedling survival for seven co‐occurring Great Basin plant taxa growing with invasive grass
Source: Ecol Evol. 2022 Apr 30;12(5):e8870. doi: 10.1002/ece3.8870 (PMC9055296; doi:10.1002/ece3.8870)
Supplement: Supplementary file 5 — Table S3 [file ECE3-12-e8870-s003.pdf]

Table S3. Pearson correlation coefficients among scaled abiotic variables for all seed collection sites. Abiotic variables follow the same acronyms as Table S2 legend.

	Elev.	Slope	Nor.	East.	Ht. ld.	SAWC	MAT	Ppt. s.	Min. VPD	AET	SDAET
**SDAET**											
**AET**											0.29
**Min. VPD**										-0.04	0.15
**Ppt. s.**									-0.19	-0.16	0.11
**MAT**								0.45*	0.53**	-0.24	0.17
**SAWC**							-0.27	-0.26	-0.18	0.32'	-0.09
**Ht. ld.**						0.31	-0.24	-0.37'	0.02	0.31	-0.05
**East.**					0.42*	0.28	0.04	-0.20	0.02	0.29	-0.03
**Nor.**				-0.05	0.60***	-0.11	-0.22	-0.30	0.04	0.12	0.19
**Slope**			0.01	0.09	-0.28	-0.42*	0.05	0.19	0.26	0.14	-0.00
**Elev.**		0.57**	0.07	0.12	0.16	-0.09	-0.51**	-0.27	0.12	0.26	-0.15
**MAP**	0.29	0.56**	0.06	0.04	-0.12	-0.08	0.00	0.54**	-0.10	0.33'	0.32'
