# Supplementary material for: Phenotypes and environment predict seedling survival for seven co‐occurring Great Basin plant taxa growing with invasive grass
Source: Ecol Evol. 2022 Apr 30;12(5):e8870. doi: 10.1002/ece3.8870 (PMC9055296; doi:10.1002/ece3.8870)
Supplement: Supplementary file 6 — Table S4 [file ECE3-12-e8870-s004.pdf]

Table S4. Sample size, number of seedlings emerged, and timing of seedling trait description for each taxon. 100 individuals per collection site were planted for each taxon and each harvest age with two exceptions: 85 for *Elymus* spp. and 150 for *A. thurberianum* due to markedly high and low emergence, respectively.

Taxa	No. Planted	No. Harvested (% emerged)	Seedling Age(s) at Time of Harvest
A. tridentata	3,200	1,273 (40%)	10- and 35-days†
C. douglasii	3,200	414 (13%)	15- and 40-days†
Elymus spp.	1,360	1,135 (84%)	10-days‡
E. nauseosa	3,200	801 (25%)	40- and 60-days†
Erigeron spp.	4,200	968 (23%)	15- and 35-days†
P. secunda	2,400	1,242 (52%)	35-days‡
A. thurberianum	3,150	1,345 (43%)	10-days‡
Total	21,060	7,178 (34%)	

† Shrubs and forbs were measured at two different ages, as the developmental stage with the strongest relationship to plant performance has not previously been described. We picked these timeframes based on observations of plant growth rates and aimed to harvest plants when they were first initiating secondary root development (early) and when plants were reaching the bottoms of their containers (late).
‡ Harvest time based on Leger et al., 2019
