# Supplementary material for: Phenotypes and environment predict seedling survival for seven co‐occurring Great Basin plant taxa growing with invasive grass
Source: Ecol Evol. 2022 Apr 30;12(5):e8870. doi: 10.1002/ece3.8870 (PMC9055296; doi:10.1002/ece3.8870)
Supplement: Supplementary file 7 — Table S5 [file ECE3-12-e8870-s005.pdf]

Table S5: Pearson's correlations among scaled trait values for all taxa at each harvesting time, for the following seed and seedling characteristics: average diameter (Ave. diam.), emergence timing/days to emergence (Days to emer.), fine root length (FRL), root mass, root mass ratio (RMR), seed weight (Seed wt.), and specific root length (SRL).

***Artemisia tridentata* (10 days)**

	Ave. diam.	Days to emer.	CV Days to emer.	FRL	Root mass	CV Root mass	RMR	Seed wt.
**Days to emer.**	0.72	-						
**CV Days to emer.**	0.12	0.40	-					
**FRL**	-0.07	-0.41	-0.20	-				
**Root mass**	-0.46	-0.47	0.24	0.55	-			
**CV Root mass**	0.18	0.32	0.12	-0.31	-0.49	-		
**RMR**	0.34	0.28	0.54	-0.13	0.30	-0.34	-	
**Seed wt.**	-0.47	-0.54	0.07	0.38	0.88	-0.55	0.28	-
**SRL**	0.06	0.03	-0.36	-0.14	-0.72	0.46	-0.63	-0.55

***Artemisia tridentata* (35 days)**

	Ave. diam.	Days to emer.	CV Days to emer.	FRL	Root mass	CV Root mass	RMR	Seed wt.
**Days to emer.**	-0.82	-						
**CV Days to emer.**	-0.16	0.51	-					
**FRL**	0.12	-0.53	-0.63	-				
**Root mass**	0.88	-0.86	-0.31	0.36	-			
**CV Root mass**	-0.60	0.74	0.26	-0.39	-0.75	-		
**RMR**	0.64	-0.57	-0.33	0.24	0.68	-0.55	-	
**Seed wt.**	0.51	-0.43	0.12	-0.12	0.61	-0.64	0.15	-
**SRL**	-0.84	0.63	-0.05	0.14	-0.83	0.73	-0.54	-0.81

***Chaenactis douglasii* (15 days)**

	Ave. diam.	Days to emer.	CV Days to emer.	FRL	Root mass	CV Root mass	RMR	Seed wt.
**Days to emer.**	0.36	-						
**CV Days to emer.**	0.32	0.30	-					
**FRL**	-0.24	0.01	-0.62	-				
**Root mass**	0.02	0.33	-0.21	0.40	-			
**CV Root mass**	0.12	-0.43	0.38	-0.53	-0.74	-		
**RMR**	-0.25	0.38	-0.51	0.56	0.76	-0.86	-	
**Seed wt.**	0.11	0.13	0.11	-0.27	0.65	-0.22	0.22	-
**SRL**	0.14	-0.44	-0.21	0.17	-0.67	0.63	-0.55	-0.65

Chaenactis douglasii **(40 days)**

	Ave. diam.	Days to emer.	CV Days to emer.	FRL	Root mass	CV Root mass	RMR	Seed wt.
**Days to emer.**	0.84	-						
**CV Days to emer.**	0.56	0.68	-					
**FRL**	-0.62	-0.64	-0.32	-				
**Root mass**	0.08	-0.20	0.05	0.49	-			
**CV Root mass**	0.55	0.70	0.70	-0.56	-0.36	-		
**RMR**	-0.34	-0.42	-0.22	0.60	0.58	-0.71	-	
**Seed wt.**	0.52	0.28	0.09	-0.46	0.22	0.12	0.08	-
**SRL**	0.38	0.60	0.43	-0.36	-0.59	0.79	-0.75	-0.22

Elymus **spp. (10 days)**

	Ave. diam.	Days to emer.	CV Days to emer.	FRL	Root mass	CV Root mass	RMR	Seed wt.
**Days to emer.**	-0.39	-						
**CV Days to emer.**	-0.22	0.60	-					

**FRL**	0.18	-0.51	-0.21	-				
**Root mass**	0.81	-0.54	-0.24	0.68	-			
**CV Root mass**	0.10	0.32	0.24	-0.57	-0.11	-		
**RMR**	-0.09	-0.39	-0.16	0.37	0.05	-0.64	-	
**Seed wt.**	0.61	-0.56	-0.41	0.37	0.68	-0.05	-0.01	-
**SRL**	-0.91	0.26	0.13	-0.14	-0.79	-0.30	0.35	-0.70

***Ericameria nauseosa* (40 days)**

	**Ave. diam.**	**Days to emer.**	**CV Days to emer.**	**FRL**	**Root mass**	**CV Root mass**	**RMR**	**Seed wt.**
**Days to emer.**	-0.32	-						
**CV Days to emer.**	0.05	0.43	-					
**FRL**	-0.36	0.22	-0.42	-				
**Root mass**	-0.03	0.21	-0.37	0.92	-			
**CV Root mass**	-0.10	0.07	0.25	-0.58	-0.58	-		
**RMR**	-0.46	0.48	-0.26	0.69	0.55	-0.61	-	
**Seed wt.**	0.08	-0.10	-0.29	0.70	0.76	-0.32	0.10	-
**SRL**	-0.81	0.11	-0.07	0.02	-0.29	0.42	0.05	-0.16

***Ericameria nauseosa* (60 days)**

	**Ave. diam.**	**Days to emer.**	**CV Days to emer.**	**FRL**	**Root mass**	**CV Root mass**	**RMR**	**Seed wt.**
**Days to emer.**	0.56	-						
**CV Days to emer.**	0.47	0.44	-					
**FRL**	-0.42	-0.05	-0.09	-				
**Root mass**	-0.19	-0.01	-0.07	0.93	-			
**CV Root mass**	-0.17	-0.26	0.31	-0.48	-0.51	-		
**RMR**	-0.17	0.26	-0.42	0.17	0.12	-0.57	-	
**Seed wt.**	-0.32	-0.22	-0.13	0.88	0.91	-0.45	0.09	-

**SRL**	-0.60	-0.22	-0.26	-0.10	-0.40	0.40	0.09	-0.29

Erigeron **spp. (15 days)**

	**Ave. diam.**	**Days to emer.**	**CV Days to emer.**	**FRL**	**Root mass**	**CV Root mass**	**RMR**	**Seed wt.**
**Days to emer.**	-0.65	-						
**CV Days to emer.**	-0.55	0.77	-					
**FRL**	-0.66	0.26	0.36	-				
**Root mass**	0.81	-0.52	-0.39	-0.61	-			
**CV Root mass**	-0.59	0.53	0.36	0.18	-0.25	-		
**RMR**	0.46	-0.45	-0.39	-0.18	0.28	-0.60	-	
**Seed wt.**	0.75	-0.52	-0.39	-0.63	0.97	-0.16	0.14	-
**SRL**	-0.89	0.67	0.34	0.55	-0.82	0.59	-0.37	-0.79

Erigeron **spp. (35 days)**

	**Ave. diam.**	**Days to emer.**	**CV Days to emer.**	**FRL**	**Root mass**	**CV Root mass**	**RMR**	**Seed wt.**
**Days to emer.**	-0.11	-						
**CV Days to emer.**	0.27	0.47	-					
**FRL**	-0.80	-0.24	-0.61	-				
**Root mass**	0.83	-0.34	-0.12	-0.41	-			
**CV Root mass**	-0.28	-0.20	-0.63	0.59	0.11	-		
**RMR**	0.72	0.12	0.07	-0.45	0.76	0.07	-	
**Seed wt.**	0.93	0.03	0.35	-0.85	0.76	-0.42	0.69	-
**SRL**	-0.83	-0.03	-0.52	0.81	-0.64	0.43	-0.54	-0.83

Poa secunda **(35 days)**

	**Ave. diam.**	**Days to emer.**	**CV Days to emer.**	**FRL**	**Root mass**	**CV Root mass**	**RMR**	**Seed wt.**
**Days to emer.**	-0.25	-						

**CV Days to emer.**	-0.12	0.87	-					
**FRL**	0.24	-0.39	-0.10	-				
**Root mass**	0.61	-0.41	-0.09	0.89	-			
**CV Root mass**	-0.48	0.24	0.25	-0.14	-0.35	-		
**RMR**	0.28	0.34	0.40	0.26	0.31	-0.07	-	
**Seed wt.**	0.47	-0.23	0.02	0.31	0.47	-0.24	0.17	-
**SRL**	-0.83	0.06	-0.11	-0.42	-0.71	0.47	-0.52	-0.42

***Achnatherum thurberianum* (10 days)**

	**Ave. diam.**	**Days to emer.**	**CV Days to emer.**	**FRL**	**Root mass**	**CV Root mass**	**RMR**	**Seed wt.**
**Days to emer.**	0.37	-						
**CV Days to emer.**	0.68	0.40	-					
**FRL**	-0.13	-0.32	0.00	-				
**Root mass**	0.43	-0.30	0.21	0.51	-			
**CV Root mass**	-0.33	0.03	-0.31	-0.35	-0.23	-		
**RMR**	0.34	-0.12	0.11	-0.01	0.62	-0.22	-	
**Seed wt.**	0.00	-0.22	-0.26	0.18	0.41	0.00	0.43	-
**SRL**	-0.69	-0.27	-0.45	0.39	-0.44	0.14	-0.63	-0.06
