# Supplementary material for: Phenotypes and environment predict seedling survival for seven co‐occurring Great Basin plant taxa growing with invasive grass
Source: Ecol Evol. 2022 Apr 30;12(5):e8870. doi: 10.1002/ece3.8870 (PMC9055296; doi:10.1002/ece3.8870)
Supplement: Supplementary file 8 — Table S6 [file ECE3-12-e8870-s008.pdf]

Table S6: Summary of linear mixed effects models for assessing trait differentiation among seed sources within each taxon. Test statistics, significance, model fits from generalized linear mixed effect models, means, and ranges of seven seed and seedling traits measured in all seed sources for each taxon. Values given are Chi-square result (χ^2) from likelihood ratio tests, p-values (* = p < 0.05, ** = p <0.01, *** = p < 0.001), conditional model fits (R^2), means and standard error (SE), and ranges from the populations with the lowest and highest trait values (n=160-1219). Trait variables follow the same acronyms as Table S5. †Reported F-value for linear model in place of Chi-square result given lack of random block effect in seed weight data.

AR10	**Trait**	**χ^2**	**R^2**	**Mean (SE)**	**Range of collection sites**
	Ave. diam. (mm)	24.2	0.15	0.41 (0.01)	0.36 (0.02) - 0.49 (0.06)
	Days to emer.	79.6***	0.12	10.64 (0.38)	7.8 (0.29) - 15.93 (2.85)
	FRL (cm)	26.4*	0.07	15.78 (0.28)	13.22 (0.9) - 18.04 (1.49)
	RMR	40.4***	0.07	0.28 (0.003)	0.25 (0.01) - 0.33 (0.03)
	Root mass (mg)	99.8***	0.15	0.34 (0.01)	0.23 (0.01) - 0.5 (0.03)
	Seed wt. (mg)	*F* = 60.7***	0.85	0.004 (<0.001)	0.003 (<0.001) - 0.006 (<0.001)
	SRL (mg/cm)	58.1***	0.13	105.35 (8.17)	56.64 (5.89) - 157.87 (60.86)
AR35	**Trait**	**χ^2**	**R^2**	**Mean (SE)**	**Range of populations**
	Ave. diam. (mm)	41.6***	0.11	0.4 (0.002)	0.36 (0.005) - 0.43 (0.03)
	Days to emer.	47.9***	0.13	9.69 (0.41)	7.51 (0.53) - 16.56 (4.2)
	FRL (cm)	40.8***	0.15	141.22 (2.29)	114.22 (5.49) - 165.79 (13.66)
	RMR	71.4***	0.19	0.47 (0.003)	0.41 (0.01) - 0.52 (0.02)
	Root mass (mg)	41.4***	0.24	16.43 (0.38)	10.11 (0.87) - 18.92 (2.1)
	Seed wt. (mg)†	*F* = 60.7***	0.85	0.004 (<0.001)	0.003 (<0.001) - 0.006 (<0.001)
	SRL (mg/cm)	132.1***	0.37	13.62 (0.26)	11.17 (0.4) - 18.78 (2.43)
CH15	**Trait**	**χ^2**	**R^2**	**Mean (SE)**	**Range of populations**
	Ave. diam. (mm)	19.2	0.08	0.38 (0.01)	0.31 (0.02) - 0.43 (0.11)
	Days to emer.	13.7	0.09	17.42 (0.37)	15.35 (0.5) - 20.5 (3.3)
	FRL (cm)	59.4***	0.20	24.34 (0.63)	16.37 (1.22) - 32.14 (3.96)
	RMR	26.1*	0.13	0.36 (0.005)	0.3 (0.01) - 0.45 (0.06)
	Root mass (mg)	62.0***	0.22	0.87 (0.02)	0.56 (0.05) - 1.23 (0.19)
	Seed wt. (mg)†	*F* = 34.7***	0.77	0.02 (<0.001)	0.01 (<0.001) - 0.02 (<0.001)
	SRL (mg/cm)	50.4***	0.19	41.96 (1.88)	28.73 (2.15) - 84.36 (25.71)
CH40	**Trait**	**χ^2**	**R^2**	**Mean (SE)**	**Range of populations**
	Ave. diam. (mm)	26.2*	0.32	0.39 (0.01)	0.32 (0.01) - 0.54 (0.25)
	Days to emer.	34.4**	0.33	16.48 (0.64)	11.14 (0.67) - 38 (18)
	FRL (cm)	32.5**	0.30	201.43 (6.49)	99.5 (8.97) - 253.08 (88.75)
	RMR	37.1**	0.27	0.52 (0.01)	0.36 (0.01) - 0.57 (0.15)
	Root mass (mg)	31.9**	0.39	14.61 (0.7)	6.96 (1.34) - 19.31 (10.01)
	Seed wt. (mg)†	*F* = 34.7***	0.77	0.02 (<0.001)	0.01 (<0.001) - 0.02 (<0.001)

	SRL (mg/cm)	51.7***	0.42	20.97 (0.99)	14.16 (0.74) - 41.89 (31.97)
EL10	**Trait**	χ^2	R^2	**Mean (SE)**	**Range of populations**
	Ave. diam. (mm)	83.1***	0.13	0.32 (0.002)	0.3 (0.01) - 0.36 (0.01)
	Days to emer.	70.9***	0.43	10.29 (0.1)	9.32 (0.28) - 11.65 (0.72)
	FRL (cm)	89.2***	0.12	17.37 (0.24)	13.57 (0.65) - 22.04 (1.39)
	RMR	38.5**	0.29	0.5 (0.003)	0.48 (0.01) - 0.53 (0.01)
	Root mass (mg)	282.4***	0.23	1.8 (0.02)	1.19 (0.05) - 2.57 (0.14)
	Seed wt. (mg)†	*F* = 14.9***	0.83	0.03 (<0.001)	0.02 (<0.001) - 0.04 (0.002)
	SRL (mg/cm)	110.2***	0.17	11.86 (0.12)	9.25 (0.35) - 13.84 (0.62)
EG15	**Trait**	χ^2	R^2	**Mean (SE)**	**Range of populations**
	Ave. diam. (mm)	125.4***	0.27	0.37 (0.01)	0.25 (0.01) - 0.47 (0.09)
	Days to emer.	78.2***	0.22	8.54 (0.22)	6.67 (0.28) - 14 (4.5)
	FRL (cm)	237.8***	0.41	28.06 (0.69)	14.39 (1.24) - 43.06 (21.32)
	RMR	19.5	0.14	0.46 (0)	0.38 (0.01) - 0.49 (0.1)
	Root mass (mg)	456.5***	0.60	1.55 (0.05)	0.45 (0.06) - 3.14 (1.33)
	Seed wt. (mg)†	*F* = 31.1***	0.70	0.005 (<0.001)	<0.001 (<0.001) - 0.01 (<0.001)
	SRL (mg/cm)	116.5***	0.69	33.66 (1.24)	11.31 (0.99) - 86.1 (32.83)
EG35	**Trait**	χ^2	R^2	**Mean (SE)**	**Range of populations**
	Ave. diam. (mm)	378.9***	0.45	0.36 (0.005)	0.28 (0.01) - 0.49 (0.04)
	Days to emer.	76.1***	0.12	8.96 (0.23)	7.2 (0.28) - 17.1 (8.88)
	FRL (cm)	331.8***	0.46	82.55 (2.12)	37.69 (3.06) - 154.81 (27.24)
	RMR	374.7***	0.47	0.56 (0.004)	0.5 (0.01) - 0.67 (0.03)
	Root mass (mg)	154.7***	0.36	8.39 (0.22)	5.11 (0.34) - 13.69 (2.06)
	Seed wt. (mg)†	*F* = 31.1***	0.70	0.005 (<0.001)	<0.001 (<0.001) - 0.01 (<0.001)
	SRL (mg/cm)	963.3***	0.70	15.63 (0.61)	5.41 (0.4) - 38.89 (11.76)
EC40	**Trait**	χ^2	R^2	**Mean (SE)**	**Range of populations**
	Ave. diam. (mm)	33.3**	0.19	0.31 (0.002)	0.28 (0.01) - 0.35 (0.03)
	Days to emer.	88.5***	0.26	35.01 (1.17)	22.96 (1.19) - 53.13 (8.87)
	FRL (cm)	82.8***	0.41	20.01 (0.82)	6.33 (1.93) - 28.65 (3.72)
	RMR	41.6***	0.43	0.41 (0.01)	0.31 (0.01) - 0.47 (0.07)
	Root mass (mg)	151.3***	0.52	1.72 (0.06)	0.77 (0.12) - 2.61 (0.29)
	Seed wt. (mg)†	*F* = 83.0***	0.89	0.01 (<0.001)	0.005 (<0.001) - 0.02 (<0.001)
	SRL (mg/cm)	56.1***	0.27	14.34 (0.33)	8.43 (0.53) - 20.02 (3.25)
EC60	**Trait**	χ^2	R^2	**Mean (SE)**	**Range of populations**
	Ave. diam. (mm)	34.8**	0.13	0.3 (0.002)	0.28 (0.004) - 0.32 (0.03)
	Days to emer.	98.5***	0.25	28.62 (1.19)	15.08 (0.84) - 52.25 (17.5)
	FRL (cm)	83.5***	0.22	78.45 (2.06)	54.16 (5.51) - 104.66 (13.92)
	RMR	36.9**	0.09	0.57 (0.004)	0.5 (0.01) - 0.6 (0.04)
	Root mass (mg)	93.3***	0.22	8.26 (0.21)	5.09 (0.51) - 11.5 (1.43)
	Seed wt. (mg)†	*F* = 83.0***	0.89	0.01 (<0.001)	0.005 (<0.001) - 0.02 (<0.001)

	SRL (mg/cm)	38.5***	0.18	11.4 (0.17)	9.76 (0.32) - 13.87 (2)
PO35	**Trait**	**χ^2**	**R^2**	**Mean (SE)**	**Range of populations**
	Ave. diam. (mm)	137.8***	0.25	0.24 (0.001)	0.22 (0.003) - 0.26 (0.01)
	Days to emer.	56.6***	0.19	17.82 (0.26)	15 (0.77) - 28.75 (15.09)
	FRL (cm)	96.1***	0.27	23.15 (0.49)	15.64 (1.14) - 30.36 (8.36)
	RMR	52.8***	0.18	0.37 (0.003)	0.32 (0.01) - 0.4 (0.03)
	Root mass (mg)	138.0***	0.29	1.53 (0.04)	0.84 (0.09) - 2.27 (0.6)
	Seed wt. (mg)†	F = 18.6***	0.64	0.005 (<0.001)	0.004 (<0.001) - 0.007 (<0.001)
	SRL (mg/cm)	140.0***	0.28	20.17 (0.37)	14.02 (0.45) - 25.99 (6.04)
AC10	**Trait**	**χ^2**	**R^2**	**Mean (SE)**	**Range of populations**
	Ave. diam. (mm)	42.1**	0.35	0.32 (0.002)	0.28 (0.01) - 0.34 (0.02)
	Days to emer.	113.5***	0.13	22.97 (0.72)	16.13 (1.35) - 39.96 (6)
	FRL (cm)	46.1***	0.41	12.25 (0.29)	8.62 (0.72) - 15.6 (2.11)
	RMR	42.1**	0.10	0.33 (0.002)	0.28 (0.01) - 0.36 (0.02)
	Root mass (mg)	121.2***	0.20	1.05 (0.02)	0.78 (0.03) - 1.42 (0.26)
	Seed wt. (mg)†	F = 37.4***	0.78	0.03 (<0.001)	0.02 (<0.001) - 0.05 (0.002)
	SRL (mg/cm)	52.1***	0.34	13.53 (0.23)	10.99 (0.49) - 17.48 (3.18)
