# Supplementary material for: Phenotypes and environment predict seedling survival for seven co‐occurring Great Basin plant taxa growing with invasive grass
Source: Ecol Evol. 2022 Apr 30;12(5):e8870. doi: 10.1002/ece3.8870 (PMC9055296; doi:10.1002/ece3.8870)
Supplement: Supplementary file 9 — Table S7 [file ECE3-12-e8870-s006.pdf]

Table S7. Summary of mutli-taxon linear models for assessing potential consistency in trait-environment associations across taxa. Relationships between traits and environment across all taxa with each seedling trait as a response variable (a-h). Values reported are from linear regression models, and include the F-test statistics, the significance of differences, and model fits; p values <0.05 are in bold.

	a) Root mass		b) Seed wt.		c) SRL		d) Days to emerge	
	F	*p*	*F*	*p*	*F*	*p*	*F*	*p*
AET	0.9	0.347	10.4	**0.001**	0.0	0.892	4.1	**0.043**
East.	0.5	0.481	4.2	**0.044**	0.7	0.393	0.2	0.683
Elev.	0.0	0.949	0.1	0.767	0.1	0.704	0.2	0.665
Ht. ld.	1.3	0.252	6.8	**0.009**	0.5	0.490	0.0	0.967
MAP	1.4	0.237	7.3	**0.007**	0.5	0.465	0.0	0.901
MAT	0.0	0.871	0.0	0.900	0.0	0.952	0.5	0.486
Min. VPD	0.1	0.743	0.0	0.925	0.0	0.853	0.4	0.517
Nor.	0.0	0.955	3.4	0.068	0.0	0.871	0.0	0.868
Ppt. s.	0.1	0.791	16.1	**<0.001**	0.0	0.988	0.2	0.660
SAWC	0.0	0.947	0.1	0.718	0.0	0.879	4.8	**0.029**
SDAET	0.0	0.856	1.9	0.170	0.0	0.940	2.0	0.157
Slope	0.5	0.503	0.0	0.862	0.8	0.370	0.3	0.603
Age	1468.1	**<0.001**	-	-	216.8	**<0.001**	1.4	0.245
Species	273.2	**<0.001**	534.5	**<0.001**	83.5	**<0.001**	103.8	**<0.001**
R^2	0.88		0.91		0.63		0.70	
	e) RMR		f) Avg. diam.		g) CV Days to emerge		h) CV Root mass	
AET	0.3	0.579	1.2	0.273	0.1	0.717	0.9	0.342
East.	4.6	**0.032**	1.1	0.287	3.2	0.073	2.5	0.113
Elev.	0.2	0.636	0.0	0.855	0.4	0.541	0.1	0.774
Ht. ld.	3.1	0.077	4.1	**0.043**	0.3	0.586	3.4	0.067
MAP	0.1	0.797	2.7	0.100	1.2	0.265	2.9	0.088
MAT	0.9	0.332	0.5	0.479	8.7	**0.003**	3.6	0.060
Min. VPD	2.1	0.150	0.6	0.443	18.1	**<0.001**	3.0	0.084
Nor.	2.6	0.109	0.7	0.411	0.1	0.801	1.2	0.281
Ppt. s.	1.4	0.241	0.3	0.612	3.2	0.074	2.2	0.139
SAWC	0.9	0.355	0.8	0.385	6.0	**0.015**	0.5	0.494
SDAET	0.5	0.496	0.6	0.431	4.9	**0.027**	1.1	0.291
Slope	1.0	0.330	0.7	0.420	14.4	**<0.001**	0.6	0.444
Age	892.6	**<0.001**	0.1	0.717	11.2	**<0.001**	1.7	0.187
Species	382.6	**<0.001**	164.6	**<0.001**	90.6	**<0.001**	32.8	**<0.001**
R^2	0.90		0.78		0.65		0.49	
