# Supplementary material for: Phenotypes and environment predict seedling survival for seven co‐occurring Great Basin plant taxa growing with invasive grass
Source: Ecol Evol. 2022 Apr 30;12(5):e8870. doi: 10.1002/ece3.8870 (PMC9055296; doi:10.1002/ece3.8870)
Supplement: Supplementary file 10 — Table S8 [file ECE3-12-e8870-s002.pdf]

Table S8. All trait-environment correlations for all taxa. Values indicate Pearson's correlation coefficient and color indicates magnitude of model correlation coefficient, brightest green to red are -1.0 to 1.0 and step down in color according to quartiles. Abiotic variables follow the same acronyms as Table S2.

Average diameter

	CH15	CH40	EG15	EG35	EL10	PO35	AC10	AR10	AR35	EC40	EC60
AET	0.12	-0.02	0.09	-0.17	0.00	-0.17	-0.16	0.18	-0.45	0.15	0.30
East.	-0.33	-0.07	-0.07	-0.25	-0.02	0.05	0.05	0.13	-0.13	-0.01	0.32
Elev.	0.14	-0.01	-0.05	-0.11	-0.15	0.43	-0.17	0.67	-0.85	-0.24	-0.28
Ht. ld.	0.35	-0.06	-0.47	-0.65	-0.17	-0.09	0.03	0.28	-0.39	-0.28	0.33
MAT	-0.26	0.19	0.21	0.34	0.35	-0.38	-0.04	-0.59	0.37	-0.16	-0.04
MAP	0.17	0.35	0.48	0.38	0.34	-0.03	0.15	0.24	-0.60	-0.04	-0.01
Min. VPD	0.18	-0.08	-0.08	-0.05	0.04	-0.29	-0.07	-0.12	-0.39	-0.39	-0.17
Nor.	0.39	0.06	-0.40	-0.42	-0.06	-0.10	-0.11	-0.09	-0.17	-0.25	0.36
Ppt. s.	0.10	0.37	0.35	0.38	0.40	-0.08	0.30	-0.07	-0.01	-0.09	-0.27
Slope	-0.11	0.33	0.39	0.32	0.00	0.18	-0.29	0.26	-0.72	-0.09	-0.21
SAWC	-0.01	-0.25	0.10	-0.11	0.09	-0.18	0.14	0.30	0.05	0.43	0.43
SDAET	0.10	0.08	0.16	0.08	0.29	-0.57	-0.02	-0.24	0.09	-0.30	0.14

Days to emergence

	CH15	CH40	EG15	EG35	EL10	PO35	AC10	AR10	AR35	EC40	EC60
AET	0.17	0.00	-0.34	0.61	-0.38	0.07	-0.19	0.35	0.34	-0.27	0.04
East.	0.15	0.04	0.17	0.34	-0.03	0.14	-0.26	0.20	0.34	-0.17	-0.22
Elev.	0.23	-0.14	0.06	0.70	0.19	-0.28	0.06	0.75	0.73	0.22	-0.08
Ht. ld.	0.31	0.07	0.27	0.53	0.08	0.21	-0.27	0.27	0.58	-0.09	0.07
MAT	-0.16	0.18	-0.15	-0.32	-0.28	0.36	-0.11	-0.71	-0.35	-0.25	-0.30
MAP	0.09	0.14	-0.36	0.48	-0.23	-0.10	0.32	0.00	0.43	-0.26	-0.08
Min. VPD	0.20	0.01	-0.18	0.38	-0.16	0.32	-0.16	-0.10	0.50	-0.20	-0.10
Nor.	0.15	0.24	0.12	0.43	-0.18	0.12	-0.28	0.10	0.30	-0.11	0.26
Ppt. s.	-0.07	0.19	-0.07	-0.15	-0.04	0.10	0.26	-0.43	-0.17	-0.13	-0.18
Slope	0.04	0.31	-0.30	0.44	-0.15	-0.14	0.06	0.23	0.44	-0.22	-0.17
SAWC	0.00	-0.14	0.10	0.02	0.12	0.01	0.17	0.26	0.07	0.03	0.17
SDAET	0.08	-0.04	-0.38	-0.12	-0.34	0.08	0.24	-0.43	0.14	-0.01	0.10

CV Days to emergence

	CH15	CH40	EG15	EG35	EL10	PO35	AC10	AR10	AR35	EC40	EC60
AET	-0.13	-0.02	-0.28	0.46	-0.51	0.17	-0.18	0.31	-0.14	-0.09	0.35
East.	-0.13	0.21	0.13	0.08	-0.24	0.14	0.00	0.19	0.29	-0.30	-0.03
Elev.	0.24	0.01	0.05	0.54	-0.07	-0.14	0.09	-0.02	0.22	0.39	-0.08
Ht. ld.	-0.03	0.33	0.26	0.05	-0.09	0.22	0.09	0.25	0.50	-0.18	-0.01
MAT	-0.38	-0.28	-0.39	-0.26	0.28	0.16	-0.12	-0.44	0.11	-0.57	-0.30
MAP	-0.16	-0.06	-0.70	0.23	0.05	0.09	0.08	-0.19	-0.10	-0.35	0.15
Min. VPD	0.01	-0.32	-0.16	0.25	0.19	0.21	0.06	-0.23	0.67	-0.21	-0.50
Nor.	-0.21	0.24	0.09	0.00	-0.11	0.08	-0.06	0.18	0.37	-0.15	0.06
Ppt. s.	-0.22	-0.03	-0.49	-0.36	0.33	0.12	0.11	-0.40	-0.20	-0.38	-0.06
Slope	0.04	-0.09	-0.58	0.31	-0.04	-0.15	-0.18	-0.35	-0.02	-0.08	-0.02
SAWC	0.26	0.14	0.29	-0.13	-0.21	0.08	-0.07	0.39	-0.29	-0.17	0.33
SDAET	-0.52	-0.30	-0.40	0.05	0.14	0.16	-0.07	-0.09	0.20	-0.10	-0.19

Fine root length

	CH15	CH40	EG15	EG35	EL10	PO35	AC10	AR10	AR35	EC40	EC60
AET	0.40	-0.06	0.28	-0.19	0.28	0.12	0.23	-0.36	-0.14	-0.26	-0.16
East.	0.21	0.31	0.28	0.30	-0.26	-0.15	0.13	0.29	-0.53	-0.30	-0.37
Elev.	0.15	0.42	-0.05	-0.27	-0.18	-0.13	-0.06	-0.44	-0.21	-0.19	-0.03
Ht. ld.	0.08	0.06	0.68	0.35	-0.11	-0.25	0.38	-0.03	-0.37	-0.22	-0.36
MAT	0.36	-0.36	-0.32	-0.06	0.11	0.00	-0.05	0.65	0.06	0.47	0.34
MAP	0.35	-0.31	-0.28	-0.54	0.21	0.51	0.00	0.21	0.07	0.46	0.58
Min. VPD	0.30	0.03	0.04	-0.19	0.09	-0.26	0.13	-0.07	-0.33	0.03	-0.02
Nor.	0.24	-0.42	0.37	0.18	0.31	-0.07	0.39	-0.26	-0.08	-0.02	-0.25
Ppt. s.	0.13	-0.45	-0.37	-0.22	0.07	0.38	-0.17	0.59	0.40	0.74	0.81
Slope	0.43	0.02	-0.44	-0.43	0.05	0.12	-0.39	-0.13	0.10	0.18	0.30
SAWC	-0.21	0.21	0.35	0.16	-0.30	-0.09	0.15	0.10	-0.18	-0.17	-0.16
SDAET	0.45	-0.31	-0.23	-0.18	0.25	0.06	0.53	0.12	0.02	0.42	0.23

Root mass

	CH15	CH40	EG15	EG35	EL10	PO35	AC10	AR10	AR35	EC40	EC60
AET	0.26	-0.18	-0.07	-0.37	0.25	-0.01	0.08	-0.26	-0.23	-0.13	-0.10
East.	0.02	0.30	-0.17	-0.06	-0.20	-0.11	0.02	0.37	-0.09	-0.34	-0.29
Elev.	0.02	0.43	-0.01	-0.30	-0.14	0.06	-0.32	-0.51	-0.81	-0.25	-0.12
Ht. ld.	-0.01	0.06	-0.63	-0.58	-0.17	-0.27	0.21	0.07	-0.34	-0.31	-0.38
MAT	0.35	-0.24	0.26	0.39	0.31	-0.20	0.06	0.59	0.38	0.35	0.37
MAP	0.67	-0.03	0.42	0.24	0.39	0.37	0.19	-0.24	-0.44	0.51	0.58
Min. VPD	0.03	0.00	0.00	-0.18	0.10	-0.38	-0.06	0.12	-0.35	-0.14	-0.08
Nor.	0.09	-0.43	-0.38	-0.46	0.07	-0.10	0.29	-0.12	-0.11	-0.11	-0.24
Ppt. s.	0.53	-0.15	0.36	0.43	0.40	0.26	0.22	0.12	0.00	0.68	0.73
Slope	0.29	0.01	0.46	0.17	0.10	0.11	-0.36	-0.40	-0.51	0.09	0.26
SAWC	-0.13	0.06	-0.07	0.08	-0.12	-0.14	-0.09	-0.08	-0.05	0.05	0.00
SDAET	0.41	-0.10	-0.02	0.02	0.38	-0.21	0.29	0.22	0.14	0.42	0.30

CV Root mass

	CH15	CH40	EG15	EG35	EL10	PO35	AC10	AR10	AR35	EC40	EC60
AET	-0.48	-0.20	0.13	-0.35	0.07	0.21	-0.05	0.16	0.36	0.33	0.36
East.	-0.27	-0.04	-0.29	0.31	0.04	0.15	-0.20	-0.16	0.07	0.50	0.03
Elev.	-0.25	-0.44	0.33	-0.27	0.26	0.17	0.01	-0.08	0.43	0.60	0.23
Ht. ld.	-0.28	0.19	0.16	0.12	-0.05	-0.13	-0.36	-0.04	0.44	0.53	0.05
MAT	-0.19	0.03	-0.34	0.28	0.10	0.29	-0.09	-0.51	-0.32	-0.37	-0.57
MAP	-0.68	-0.17	0.00	-0.09	0.25	0.34	-0.26	-0.19	0.63	0.00	-0.23
Min. VPD	-0.28	-0.22	0.01	-0.10	-0.11	0.55	-0.02	-0.46	0.17	0.25	-0.31
Nor.	-0.24	0.35	0.55	-0.07	-0.45	-0.12	-0.19	0.22	0.37	0.14	0.06
Ppt. s.	-0.25	0.07	-0.08	0.25	0.50	0.11	-0.30	-0.39	0.12	-0.48	-0.54
Slope	-0.43	-0.31	0.04	-0.15	0.25	0.46	0.11	-0.32	0.27	0.08	-0.17
SAWC	0.22	0.05	-0.26	0.10	0.09	-0.09	-0.12	0.37	0.20	0.04	0.18
SDAET	-0.42	0.05	-0.13	0.18	0.06	0.52	-0.16	-0.21	0.14	-0.08	-0.49

Root mass ratio (RMR)

	CH15	CH40	EG15	EG35	EL10	PO35	AC10	AR10	AR35	EC40	EC60
AET	0.23	0.23	-0.22	-0.30	-0.19	-0.11	-0.24	0.17	-0.06	-0.35	-0.42
East.	0.20	0.33	-0.02	-0.06	-0.44	-0.04	0.16	0.40	0.02	-0.16	-0.01
Elev.	0.01	0.25	-0.44	-0.03	-0.15	-0.20	-0.22	0.26	-0.40	-0.27	0.30
Ht. ld.	-0.02	-0.10	-0.41	-0.40	-0.09	-0.09	0.26	0.48	-0.18	-0.03	0.06
MAT	0.37	-0.16	0.48	0.37	-0.07	0.32	0.18	-0.22	-0.23	0.21	0.12
MAP	0.43	0.09	0.09	0.41	-0.10	0.00	0.11	-0.16	-0.60	-0.03	-0.11
Min. VPD	0.10	-0.20	-0.11	0.17	0.23	0.35	-0.20	0.25	-0.63	-0.07	0.50
Nor.	0.07	-0.48	-0.39	-0.07	0.46	0.08	0.23	0.13	-0.10	0.08	0.08
Ppt. s.	0.34	-0.11	0.44	0.42	-0.16	0.14	0.33	-0.37	-0.40	0.30	0.07
Slope	0.25	0.15	0.14	0.33	-0.07	0.01	-0.13	0.02	-0.55	-0.11	0.31
SAWC	-0.25	0.25	-0.03	-0.28	-0.37	-0.31	-0.24	0.16	0.26	0.09	-0.37
SDAET	0.33	-0.29	0.25	0.16	0.00	0.11	-0.04	0.03	-0.21	0.13	0.14

Seed weight

	CH15	CH40	EG15	EG35	EL10	PO35	AC10	AR10	AR35	EC40	EC60
AET	-0.03	-0.03	-0.01	-0.03	0.26	-0.28	-0.23	-0.20	-0.20	-0.13	-0.13
East.	-0.20	-0.20	-0.18	-0.19	-0.02	-0.27	-0.11	0.34	0.34	-0.34	-0.34
Elev.	-0.26	-0.26	0.13	0.11	-0.19	-0.04	-0.65	-0.43	-0.43	-0.06	-0.06
Ht. ld.	-0.25	-0.25	-0.56	-0.58	-0.15	-0.35	-0.25	-0.09	-0.09	-0.39	-0.39
MAT	0.20	0.20	0.14	0.16	0.31	-0.13	0.58	0.56	0.56	0.46	0.46
MAP	0.40	0.40	0.44	0.48	0.01	0.08	0.06	-0.26	-0.26	0.55	0.55
Min. VPD	-0.41	-0.41	0.02	0.01	0.02	-0.40	-0.08	0.09	0.09	0.07	0.07
Nor.	-0.31	-0.31	-0.31	-0.35	-0.05	-0.31	-0.06	-0.34	-0.34	-0.17	-0.17
Ppt. s.	0.54	0.54	0.28	0.32	0.31	0.29	0.54	0.05	0.05	0.75	0.75
Slope	-0.10	-0.10	0.47	0.45	0.08	-0.16	-0.14	-0.23	-0.23	0.28	0.28
SAWC	0.06	0.06	-0.07	-0.08	0.02	0.01	-0.26	-0.18	-0.18	-0.20	-0.20
SDAET	0.21	0.21	-0.03	0.01	0.03	0.08	0.34	0.13	0.13	0.44	0.44

Specific root length

	CH15	CH40	EG15	EG35	EL10	PO35	AC10	AR10	AR35	EC40	EC60
AET	-0.10	-0.20	-0.04	-0.08	-0.16	0.07	0.23	0.03	0.34	-0.18	-0.11
East.	-0.08	-0.06	0.11	-0.05	0.01	0.00	-0.02	-0.40	-0.22	0.19	-0.21
Elev.	-0.06	-0.26	-0.05	0.03	0.15	-0.17	0.08	0.01	0.66	0.44	0.28
Ht. ld.	0.03	0.13	0.49	0.28	0.18	0.06	-0.05	-0.34	0.25	0.27	0.07
MAT	-0.07	0.13	-0.05	-0.14	-0.34	0.23	0.09	-0.20	-0.45	0.12	-0.21
MAP	-0.49	-0.20	-0.24	-0.36	-0.28	-0.11	-0.32	0.17	0.62	-0.13	-0.25
Min. VPD	0.07	0.08	0.09	-0.11	0.06	0.29	0.37	-0.32	0.12	0.59	0.29
Nor.	0.15	0.39	0.53	0.25	0.26	-0.04	0.05	-0.02	0.24	0.14	0.21
Ppt. s.	-0.32	-0.02	-0.13	-0.05	-0.49	-0.14	-0.43	0.11	0.21	-0.17	-0.16
Slope	-0.25	0.05	-0.21	-0.21	-0.03	-0.07	0.00	0.27	0.56	0.24	0.07
SAWC	0.00	-0.15	-0.15	-0.09	-0.20	0.07	0.09	-0.02	0.04	-0.49	-0.46
SDAET	-0.02	-0.06	-0.06	-0.11	-0.21	0.47	0.27	-0.28	-0.08	-0.02	-0.36
