# Supplementary material for: Phenotypes and environment predict seedling survival for seven co‐occurring Great Basin plant taxa growing with invasive grass
Source: Ecol Evol. 2022 Apr 30;12(5):e8870. doi: 10.1002/ece3.8870 (PMC9055296; doi:10.1002/ece3.8870)
Supplement: Supplementary file 11 — Table S9 [file ECE3-12-e8870-s001.pdf]

Table S9. Model selection and model averaging results for generalized linear models testing effects of seed and seedling trait characteristics on survival across gardens and taxa. Significance of each individual predictor variable is indicated with * (`p<0.10, *p < 0.05, **p < 0.01, ***p < 0.001) for model selection, and coefficients from model averaging with standard errors that overlap zero (i.e., coefficients not significantly different from zero) are shown in italics. Trait variables follow the same acronyms as Table S5

**Best models**	**R^2**	**AIC**	**Δ AIC**
**(a) Survival of *A. tridentata* in greenhouse (10-day traits)**			
FRL***	0.5797	-1.0198	-
Ave. diam.’, FRL***	0.6567	-0.6209	0.3989
Days to emer., FRL***	0.6504	-0.3316	0.6882
RMR, FRL***	0.6257	0.7643	1.7841
Model averaging coefficients: 0.21 FRL, *-0.01 RMR, -0.02 Days to emer., -0.02 Ave. diam.*			
**(b) Survival of *A. tridentata* in greenhouse (35-day traits)**			
Root mass***, RMR***	0.6062	1.5737	-
Root mass ***, RMR***, CV Root mass	0.6744	2.8972	1.3235
Model averaging coefficients: 0.29 Root mass, -0.29 RMR, *0.04 CV Root mass*			
**(c) Survival of *C. douglasii* in greenhouse (15-day traits)**			
1	0	9.6070	-
FRL	0.1034	10.9379	1.3309
Root mass	0.0670	11.5736	1.9666
Model averaging coefficients: *-0.01 Root mass, -0.03 FRL*			
**(d) Survival of *C. douglasii* in greenhouse (40-day traits)**			
1	0	9.6070	-
RMR’, CV Root mass *	0.2604	11.4947	1.8877
CV Root mass	0.0699	11.5240	1.917
Model averaging coefficients: *0.06 CV Root mass, 0.04 RMR*			
**(e) Survival of *Elymus* spp. at California garden**			
Root mass***	0.4751	-10.2478	-
SRL***, RMR*	0.5274	-8.5473	1.7005
SRL***, CV Root mass*	0.5268	-8.5254	1.7224
Model averaging coefficients: *0.07 Root mass, 0.02 RMR, -0.02 CV Root mass, -0.07 SRL*			
**(f) Survival of *Elymus* spp. at Nevada garden**			
1	0	-6.2356	-
Seed wt.	0.1020	-5.0761	1.1595
Model averaging coefficients: *0.02 Seed wt.*			
**(g) Survival of *Elymus* spp. at Oregon garden**			
Seed wt.*, SRL’	0.3213	-10.0910	-
1	0	-9.9785	0.1125
Seed wt.	0.1444	-9.6402	0.4508
CV Root mass	0.1113	-8.9957	1.0953
Seed wt.**, SRL*, CV Days to emer.	0.4142	-8.4722	1.6188
Seed wt., CV Root mass	0.2431	-8.2372	1.8538
Model averaging coefficients: *0.06 Seed wt., 0.03 SRL, -0.01 CV Root mass, 0.01 CV Days to emer.*			
**(h) Survival of *E. nauseosa* in greenhouse (40-day traits)**			
Root mass***, Seed wt.***	0.5950	-5.4501	-
Root mass***, Seed wt.***, RMR’	0.6756	-4.6383	0.8118

Days to emer., Root mass***, Seed wt.***	0.6541	-3.6097	1.8404
Model averaging coefficients: -0.27 Root mass, 0.30 Seed wt., *0.03 RMR*, *0.01 Days to emer.*			
**(i) Survival of *E. nauseosa* in greenhouse (60-day traits)**			
Days to emer.***, RMR***, CV Days to emer.**	0.7167	-6.8023	-
Model averaging coefficients: -0.24 Days to emer., 0.20 RMR, 0.15 CV Days to emer.			
**(j) Survival of *Erigeron spp.* in greenhouse (15-day traits)**			
Root mass***	0.5642	9.5528	-
Root mass***, RMR’	0.6445	9.5769	0.0241
Model averaging coefficients: 0.28 Root mass, *0.06 RMR*			
**(k) Survival of *Erigeron spp.* in greenhouse (35-day traits)**			
Days to emer.***, SRL*, Ave. diam.***	0.8344	0.7146	-
Days to emer.***, Ave. diam.***	0.7675	2.3608	1.6462
Model averaging coefficients: -0.16 Days to emer., *0.13 SRL*, 0.39 Ave. diam.			
**(l) Survival of *P. secunda* at California garden**			
1	0	26.8910	-
CV Days to emer.	0.1091	27.2107	0.3197
Days to emer.	0.0770	27.9526	1.0616
Model averaging coefficients: *-0.03 Days to emer., -0.05 CV Days to emer.*			
**(m) Survival of *A. thurberianum* at California garden**			
1	0	-11.7797	-
CV Days to emer.	0.0808	-10.8033	0.9764
Model averaging coefficients: *-0.02 CV Days to emer.*			
