# Supplementary material for: Phenotypes and environment predict seedling survival for seven co‐occurring Great Basin plant taxa growing with invasive grass
Source: Ecol Evol. 2022 Apr 30;12(5):e8870. doi: 10.1002/ece3.8870 (PMC9055296; doi:10.1002/ece3.8870)
Supplement: Supplementary file 12 — Table S10 [file ECE3-12-e8870-s009.pdf]

Table S10. Model selection and model averaging results for generalized linear models testing effects of collection source environment characteristics on survival across gardens and taxa. Significance of each individual predictor variable is indicated with * (`p<0.10, *p < 0.05, **p < 0.01, ***p < 0.001) for model selection, and coefficients from model averaging with standard errors that overlap zero (i.e., coefficients not significantly different from zero) are shown in italics. Abiotic and environmental variables follow the same acronyms as Table S3.

Best models	R^2	AIC	Δ AIC
**(a) Survival of *A. tridentata* in greenhouse**			
Nor.*, MAT**, Ppt. s.**	0.7938	-4.4157	-
Ppt. s.***, Max. CWD**	0.7283	-4.3650	0.0507
MAT*, Ppt. s.**	0.7123	-3.4453	0.9704
Model averaging coefficients: 0.16 Ppt. s., *0.09 MAT, 0.03 Nor., -0.04 Max. CWD*			
**(b) Survival of *C. douglasii* in greenhouse**			
1	0	9.6070	-
Max. CWD’, SDAET*	0.3384	9.7112	0.1042
SDAET	0.1569	9.9531	0.3461
Elev., SDAET*	0.3020	10.5691	0.9621
East.	0.0936	11.1112	1.5042
Min. VPD, Max. CWD*, SDAET*	0.4464	11.2224	1.6154
Elev., Max. CWD', SDAET**	0.4420	11.3496	1.7426
MAP	0.0769	11.4034	1.7964
Elev.	0.0740	11.4541	1.8471
MAT, SDAET*	0.2568	11.5706	1.9636
Model averaging coefficients: *-0.09 SDAET, -0.02 Elev., 0.01 MAT, -0.01 MAP, 0.01 East., -0.04 Max. CWD, -0.01 Min. VPD*			
**(c) Survival of *Elymus* spp. at California garden**			
MAP*, Slope*	0.3424	-2.9288	-
1	0	-2.2803	0.6485
East.**, SAWC**, Max. CWD*, AET**	0.5854	-1.7060	1.2228
AET	0.1248	-1.5584	1.3704
East.’, AET’	0.2870	-1.5543	1.3745
MAP*, Slope*, East.	0.4393	-1.5194	1.4094
East.	0.0946	-0.9810	1.9478
Model averaging coefficients: *0.05 MAP, 0.04 AET, 0.02 SAWC, -0.02 Max. CWD, -0.04 Slope, -0.05 East.*			
**(d) Survival of *Elymus* spp. at Nevada garden**			
Slope*	0.3062	-	-
Model averaging coefficients: -0.10 Slope			
**(e) Survival of *Elymus* spp. at Oregon garden**			
Slope*	0.2368	-11.5845	-
1	0	-9.9785	1.606
Model averaging coefficients: *-0.05 Slope*			
**(f) Survival of *E. nauseosa* in greenhouse**			
Elev.**, MAT***	0.5371	-3.3118	-
Model averaging coefficients: 0.20 MAT, 0.14 Elev.			
**(g) Survival of *Erigeron* spp. in greenhouse**			

Nor.**, Ht. ld.***, SAWC***, MAT***	0.9368	-	-
Model averaging coefficients: -0.11 Nor., -0.38 Ht. ld., 0.19 SAWC, 0.16 MAT			
**(h) Survival of *P. secunda* at California garden**			
1	0	-14.2394	-
East.	0.0456	-12.7318	1.5076
AET	0.0439	-12.6874	1.5520
Min. VPD	0.0341	-12.4425	1.7969
MAT	0.0299	-12.3394	1.9000
Model averaging coefficients: *-0.01 Min. VPD, -0.01 AET, -0.01 MAT, -0.01 East*			
**(i) Survival of *A. thurberianum* at California garden**			
Slope**	0.3432	-9.2403	-
Slope***, SAWC	0.3829	-7.4631	1.7772
Slope**, MAT	0.3805	-7.3802	1.8601
Model averaging coefficients: -0.12 Slope, *-0.01 SAWC, -0.01 MAT*			
